# SCMR expert consensus statement for cardiovascular magnetic resonance of patients with a cardiac implantable electronic device

**DOI:** 10.1016/j.jocmr.2024.100995

**Published:** 2024-01-12

**Authors:** Daniel Kim, Jeremy D. Collins, James A. White, Kate Hanneman, Daniel C. Lee, Amit R. Patel, Peng Hu, Harold Litt, Jonathan W. Weinsaft, Rachel Davids, Kanae Mukai, Ming-Yen Ng, Julian A. Luetkens, Ariel Roguin, Carlos E. Rochitte, Pamela K. Woodard, Charlotte Manisty, Karolina M. Zareba, Lluis Mont, Frank Bogun, Daniel B. Ennis, Saman Nazarian, Gregory Webster, Jadranka Stojanovska

**Affiliations:** a Department of Radiology, Northwestern University Feinberg School of Medicine, Chicago, IL, USA; b Department of Radiology, Mayo Clinic, Rochester, MN, USA; c Departments of Cardiac Sciences and Diagnostic Imaging, Cummings School of Medicine, University of Calgary, Calgary, Canada; d Department of Medical Imaging, University Medical Imaging Toronto, Toronto General Hospital and Peter Munk Cardiac Centre, University of Toronto, Toronto, Canada; e Department of Medicine (Division of Cardiology), Northwestern University Feinberg School of Medicine, Chicago, IL, USA; f Cardiovascular Division, University of Virginia, Charlottesville, VA, USA; g School of Biomedical Engineering, ShanghaiTech University, Shanghai, China; h Department of Radiology, Perelman School of Medicine of the University of Pennsylvania, Philadelphia, PA, USA; i Department of Medicine (Division of Cardiology), Weill Cornell Medicine, New York, NY, USA; j SHS AM NAM USA DI MR COLLAB ADV-APPS, Siemens Medical Solutions USA, Inc., Chicago, Il, USA; k Salinas Valley Memorial Healthcare System, Ryan Ranch Center for Advanced Diagnostic Imaging, Monterey, CA, USA; l Department of Diagnostic Radiology, School of Clinical Medicine, Li Ka Shing Faculty of Medicine, The University of Hong Kong, the Hong Kong Special Administrative Region of China; m Department of Diagnostic and Interventional Radiology, University Hospital Bonn, Venusberg-Campus 1, Bonn, Germany; n Department of Cardiology, Hillel Yaffe Medical Center, Hadera and Faculty of Medicine. Technion - Israel Institute of Technology, Israel; o Heart Institute, InCor, University of São Paulo Medical School, São Paulo, SP, Brazil; p Mallinckrodt Institute of Radiology, Washington University School of Medicine in St. Louis, St. Louis, MO, USA; q Institute of Cardiovascular Science, University College London, London, UK; r Division of Cardiovascular Medicine, The Ohio State University, Columbus, OH, USA; s Cardiovascular Institute, Hospital Clínic, University of Barcelona, Catalonia, Spain; t Division of Cardiovascular Medicine, University of Michigan, Ann Arbor, MI, USA; u Department of Radiology, Stanford University, Stanford, CA, USA; v Section of Cardiac Electrophysiology, Perelman School of Medicine of the University of Pennsylvania, Philadelphia, PA, USA; w Department of Pediatrics (Cardiology), Ann & Robert H. Lurie Children’s Hospital, Chicago, IL, USA; x Department of Radiology, Grossman School of Medicine, New York University, New York, NY, USA

**Keywords:** Cardiac implantable electronic device, MR safety, Cardiovascular magnetic resonance, Guidelines

## Abstract

Cardiovascular magnetic resonance (CMR) is a proven imaging modality for informing diagnosis and prognosis, guiding therapeutic decisions, and risk stratifying surgical intervention. Patients with a cardiac implantable electronic device (CIED) would be expected to derive particular benefit from CMR given high prevalence of cardiomyopathy and arrhythmia. While several guidelines have been published over the last 16 years, it is important to recognize that both the CIED and CMR technologies, as well as our knowledge in MR safety, have evolved rapidly during that period. Given increasing utilization of CIED over the past decades, there is an unmet need to establish a consensus statement that integrates latest evidence concerning MR safety and CIED and CMR technologies. While experienced centers currently perform CMR in CIED patients, broad availability of CMR in this population is lacking, partially due to limited availability of resources for programming devices and appropriate monitoring, but also related to knowledge gaps regarding the risk-benefit ratio of CMR in this growing population. To address the knowledge gaps, this SCMR Expert Consensus Statement integrates consensus guidelines, primary data, and opinions from experts across disparate fields towards the shared goal of informing evidenced-based decision-making regarding the risk-benefit ratio of CMR for patients with CIEDs.

## 1 Introduction

There is a large body of evidence supporting use of cardiovascular magnetic resonance (CMR) for a broad array of indications due to its versatility, well-defined endpoints for cardiovascular health, and unique ability to identify tissue-based mechanisms of adverse cardiovascular remodeling to inform diagnosis, therapeutic decision-making, and clinical risk stratification [1], 2]. Patients with cardiac implantable electronic devices (CIEDs) may derive particular benefit from CMR given a high prevalence of cardiomyopathy and arrhythmia – conditions for which CMR has been shown to have particular diagnostic and prognostic utility in non-device patients [3], [4]. While the diagnostic and prognostic utility of CMR is less established for CIED patients, a growing number of “wideband” CMR pulse sequences are being developed and refined by academia and industry to increase the diagnostic yield of CMR in CIED patients. Given increasing utilization of CIED over the past decades [5], there is an unmet need to establish informed decision-making for CMR in this expanding population.

Prior to 2000, CIEDs were generally considered absolute contraindications for magnetic resonance imaging (MRI)[6]. Since the development of modern (manufactured after 2000 [7]) CIEDs with improved magnetic resonance (MR) safety profiles, several contemporary studies [8], [9], [10], [11], [12], [13] in patients with non-MR-conditional (a.k.a., MR-unlabeled or legacy) CIEDs, including during adenosine stress [14], have demonstrated that MRI can be performed with relatively low risk in patients with not only MR-conditional, but also non-MR-conditional CIEDs using specific protocols at 1.5 Tesla (T) [10]. Citing such data, the 2007 American Heart Association statement [15], 2008 European Society of Cardiology statement [16], 2017 Heart Rhythm Society (HRS) consensus statement [17], 2021 Recommendation by the International Society for Magnetic Resonance in Medicine safety committee [18], and 2021 Canadian [19] and 2022 British [20] consensus statements made recommendations for utilization of MRI in CIED patients using specific protocols at 1.5T. While experienced centers currently perform CMR in CIED patients, broad availability of CMR in this population is lacking, partially due to limited availability of resources for programming devices and appropriate monitoring, but also related to knowledge gaps regarding the risk-benefit ratio of CMR in this growing population [17].

To address these knowledge gaps, this SCMR Expert Consensus Statement integrates consensus guidelines, primary data, and opinion from experts across disparate fields (translational CMR, physics/engineering, electrophysiology, legal/risk management) towards the shared goal of informing evidenced-based decision-making regarding the risk-benefit ratio of CMR for patients with CIEDs. The key objectives of this statement include: (1) alternative imaging modalities for CIED patients; (2) technical explanations of MR safety across the lifespan (inclusive of pediatric and adult populations), CIED type (MR-conditional vs. non-MR-conditional), cardiac lead type/configuration (inclusive of endocardial, epicardial, and abandoned leads), and across different magnetic field strengths; (3) legal/risk management considerations for non-MR-conditional scenarios; (4) technical considerations for MRI pulse sequence optimization regarding image quality; and (5) clinical indications for CMR in symptomatic patients with CIEDs.

## 2 Alternative imaging modalities for CIED patients

Multiple alternative imaging modalities are available. These include, but are not limited to, single photon emission computed tomography (SPECT), positron emission tomography (PET), echocardiography, computed tomography (CT), and cardiac catheterization. Many of these alternatives have substantial limitations, especially given that a significant fraction of CIED patients have a high burden of arrhythmia. Examples of patients who may benefit from alternative modalities are those with absolute contraindications to CMR, patients who do not consent for the potential risks of CMR, and patients who are evaluated in imaging centers with insufficient expertise to conduct CMR in CIED patients.

Although a tabulation of the risks and benefits of each alternative modality is beyond the scope of this article, in brief, cardiac CT and cardiac catheterization deliver ionizing radiation, iodinated contrast agent, and are affected by metal artifacts caused by a combination of beam hardening, photon starvation, and scatter artifacts which may interfere with interpretation of results. CMR is affected by CIED-induced artifacts, primarily due to the transformer embedded in the ICD generator, to a lesser extent due to pacemaker generators, and to an even less extent due to cardiac leads [21]. In contrast, the artifact from CIED leads on CT can be extensive and can particularly impact septal image quality where the lead tip is typically implanted. Cardiac catheterization provides biplane, but not cross-sectional, imaging at most clinically-relevant doses of ionizing radiation and catheterization carries invasive risks that may not be appropriate for patients with lower pre-test probability of disease. Cardiac CT may require retrospective ECG-gating in patients with arrhythmia, which increases radiation dose. For both SPECT and PET equipped with CT, metal artifacts may interfere with attenuation correction. Echocardiography is commonly used prior to cross-sectional imaging regardless of modality, but has several limitations, including quality of right ventricular imaging and myocardial tissue characterization.

## 3 Up-to-date Evidence on MR Safety and SCMR-Endorsed Recommended MR Safety Protocols

A coordinated, team-based approach is required to optimize MR safety in patients with CIEDs. In this section we provide an overview of core requirements for implementation of safety protocols for imaging of patients with MR-conditional and non-MR-conditional CIEDs. This summary is based upon recently published Societal consensus statements [19], [20], while providing an overview of contributory studies supporting their development. The latter is not intended to serve as a comprehensive review of the literature, which has been published elsewhere [17], [22].

### 3.1 Previous studies assessing MR safety in patients with non-MR-conditional CIEDs

Justified by historic challenges of MRI in patients with non-MR-conditional CIEDs, device manufacturers have migrated over the past decade towards MR-conditional device systems. Studies evaluating specific generator-lead combinations have shown excellent safety in patients undergoing MR examinations, both in short-term [23], [24] and long-term [25] follow-up. Concurrent to these efforts, expanding evidence was provided by retrospective series [9], [12], [13], [26], [27] and prospective observational cohort studies [28], [29], [30], [31], [32], [33] supporting an acceptable safety profile when scanning non-MR-conditional devices using strict pre- and post-procedural protocols.

In a systematic review and meta-analysis performed by Shah, et al. in 2018, including 5099 patients undergoing 5908 MRI examination from 31 eligible studies, the observed complication rate was very low. No deaths were reported and only 17 (0.3%) patients reported minor symptoms. A total of 94 power-on resets were reported (1.6% of scans), however these were isolated to generators older than 2006. There were 3 lead failures reported, none directly and immediately attributable to MRI. Table 1 provides an overview of major published MRI safety studies in patients with CIED. With cumulative evidence from over 6000 patients with non-MR-conditional permanent pacemaker (PPM) or implantable cardioverter defibrillator (ICD) systems, each study has described a low rate of complications resulting in device revision or clinically relevant outcomes. Of these studies, three large prospective cohort studies delivered dominant evidence. A study published by Nazarian, et al. in 2017 reported on 1509 patients (880 PPM, 629 ICD) undergoing 2103 MRI studies at 1.5T, including pre- and post-MR device interrogations and follow-up [13]; only eight patients (0.5%) experienced a power-on reset while only 1 device had permanent reset due to near end-of-life battery; there were no clinically relevant adverse outcomes. A second prospective study published by Russo, et al. in 2017 reported on 1246 patients undergoing 1500 MRI scans at 1.5T (1000 PPM, 500 ICD); a similarly low event rate was observed, with only 1 permanent reset and no clinical events [12]. A prospective study by Gupta, et al. was published in 2020 examining MR safety outcomes in 532 patients (279 PPM, 186 ICD, 26 cardiac resynchronization therapy pacemaker [CRT-P] and 105 cardiac resynchronization therapy defibrillator [CRT-D]) undergoing 608 MRI studies at 1.5T [26]. They observed only transiently increased impedance in one lead without clinically relevant complications. Although retrospective, a large cohort study was also published in 2019 by Vuorinen examining safety outcomes following 1000 MRI scans at 1.5T in 793 patients, with similarly low rates of device or patient-related complications [28]. Finally, a study by Fluschnik et al. [34] in 2022 reported on 97 patients undergoing 132 MRI scans at 3T, no adverse events immediately after MRI.

**Table 1:** Studies reporting MR safety from scanning patients with a CIED.

First Author, Publication Year, Country	Population Size City, Country	Study Design Institution (s)	Cardiac Implantable Electronic Devices	Field Strength Sequences	MRI Scans Anatomic Regions	Outcomes	Findings
**Nazarian, et al.**[9] 2011 United States and Israel	438 patients 555 MRIs Baltimore, Maryland, USA and Haifa, Israel	2 center prospective non-randomized trial Johns Hopkins University, USA and Rambam Medical Center, Israel	Non-MR-conditional PPM (n = 237 patients) or ICD (n = 201 patients) Excluded abandoned or epicardial leads	1.5T Standard sequences	• 89 cardiac • 344 brain/spine • 79 abdomen/pelvis • 50 extremity	• Activation or inhibition of pacing • Symptoms • Device variables	• 3 patients (0.7%) had a power-on reset, although without device dysfunction during long-term follow-up • RV sensing and atrial and right and left ventricular lead impedances were reduced immediately post-MRI • At long-term follow-up, there was decreased RV sensing, decreased RV lead impedance, increased RV capture threshold and decreased battery voltage • None required device revision /reprogramming
**Camacho, et al.**[33] 2016 United States	104 patients 113 MRIs Atlanta, Georgia, USA	Single center retrospective cohort study Emory University, USA Dates of the scans were not provided	Non-MR-conditional PPM (n = 74 scans) or ICD (n = 39 scans) Abandoned or capped leads were excluded 5 patients were pacer dependent	1.5T Standard sequences	• 3 cardiac • 5 chest • 47 abdomen/pelvis • 81 brain, C/T/L spine • 3 neck • 2 extremity	• Changes in lead impedance, sensing, or thresholds • Episodes of electromagnetic interference or noise • Programming changes before or after the MRI • Patient symptoms • Abnormal device activity	• No significant changes in lead parameters • Electromagnetic noise was detected on at least 1 lead in 7.1% of studies • Patients reported transient symptoms during 3 examinations (heating at the pocket site, tingling at the pocket site, and palpitations) without complications • No abnormal device activity • No emergency termination of the MRI • All studies were diagnostic
**Nazarian, et al.**[13] 2017 United States	1509 patients 2103 MRIs Baltimore, Maryland, USA	Single center prospective observational cohort study Johns Hopkins University, USA February 2003Through January 2015	Non-MR-conditional PPM (n = 880 patients) or ICD (n = 629 patients) 137 patients with device dependence. Pacers year 1996 or later and ICDs year 2000 or later were included	1.5T Standard sequences	No cardiac scans • 257 thoracic	• Generator failure • Power-on reset • Change in pacing or sensing thresholds requiring programming changes • Battery depletion • Cardiac arrhythmia • Inhibition of pacing • Inappropriate ATP or shock • Patient symptoms	• 9 MRI scans (0.4%) in 8 patients (0.5%) had power-on reset; this was transient in all but 1 scan • Devices were manufactured between 1997-2009 -Device program failure in 1 device (less than 1 month of battery remaining) • -No long-term clinically significant events
**Russo, et al.**[12] 2017 United States	1246 patients 1500 MRIs Multiple locations in the USA	Multi-center prospective observational cohort study April 2009 through April 2014 Scripps Research Institute, USA and 19 centers in the USA	Non-MR-conditional PPM (n = 818 patients) or ICD (n = 428 patients) 1000 pacer scans 500 ICD scans	1.5T Standard sequences	No cardiac scans • 591 brain • 249 C spine • 448 L spine • 168 extremity/joint • 81 abdomen/pelvis • 172 other	• Death • Generator or lead failure requiring immediate replacement • Loss of capture (for pacer dependent patients) • New arrhythmia • Partial or full generator electrical reset	• Nodeaths • No ventricular arrhythmias • No lead failure • 6 cases of self-terminating atrial fibrillation/flutter • 6 cases of partial electrical reset • 1 ICD device programming failure due to protocol violation
Okamura, at al.[29] 2017 United States	9 patients with PPM and ICD with a nearly depleted battery 13 MRIs Rochester, Minnesota, USA	Single center retrospective observational cohort study Mayo Clinic, USA January 2008 to May 2015	8 scans with devices at ERI Non-MR-conditional PPM (n = 4 scans with a device with a nearly depleted battery)) or ICD (n = 9 scans with a device with a nearly depleted battery) Pacer dependent patients were excluded	1.5T Standard sequences	No cardiac scans • 11 head • 3 chest Some patients had both scans at the same time	• Power on reset • Elective replacement indicator (ERI) turned on • Unable to reprogram the device • All events occurred in pacemakers implanted before 2005	• 2 scans with pacers close to ERI resulted in a power on reset • 1 scan with a pacer close to ERI resulted in a power on reset during MRI and automatically changed to VVI mode • 1 scan with a pacer at ERI did not allow reprogramming
**Do, et al.**[32] 2018 United States	111 patients 111 MRIs Los Angeles, California, USA	Single center retrospective observational cohort study UCLA, USA April 2013To October 2016	Non-MR-conditional PPM (n = 12 patients), ICD (n = 73 patients), and CRT-D (n = 29 patients) 3 patients were device dependent (1 with pacer, 1 with ICD, and 1 with CRT-D) Out of 114 consecutive studies, 3 scans were stopped prematurely and excluded due to anginal chest pain, anxiety, and frequent non-sustaiend VT prior to the scan	1.5T Wideband sequences for late gadolinium enhancement (LGE)	Cardiac scans • 111 cardiac	• Clinical deterioration nor death during the scan • Generator failure requiring replacement • Lead failure requiring replacement • New-onset arrhythmia • Loss of capture in pacemaker-dependent patients Power-on reset	• No adverse CIED complications or clinical outcomes • 87% had no artifact limiting interpretation
**Shah, et al.**[135] 2018 United States	5099 patients 5908 MRIs Multiple locations	Systematic review & meta-analysis 70 studies were included in the systematic review 31 studies were included in the meta-analysis cohort	Non-MR-conditional devices (3147 RA leads, 4023 RV leads, 268 LV leads); 1440 defibrillator leads; 100 abandoned leads, 25 epicardial leads, 4 subcutaneous ICD, small number of temporary pacemakers 3692 pacer patients 1440 ICD patients, 268 LV pacing leads 551 pacer dependent patients 39 patients with AICD and device dependent	0.2T 0.5% 1.5T 2T 3T Standard sequences	No cardiac scans • 773Thoracic • 3105 head/neck • 1153 abdomen/pelvis/L spine • 402 extremity	• Deaths • Lead survival • Lead performance • Electrical reset • Inappropriate ICD shock and therapy • High voltage impedance • Patient symptoms • Battery voltage change	• No deaths1 ICD shock (inadvertently scanned at 0.2 T) • 3 Lead failures (none directly attributable to MRI) • 94 electrical resets (all devices older than 2006) • No changes in lead parameters, battery or generator performance
**Lupo, et al.**[27] 2018 Italy	120 patients 142 MRIs Milan, Italy	Single center prospective cohort study Humanitas University, Italy December 2006 to November 2014	Non-MR-conditional PPM (n = 71 scans) or ICD (n = 71 scans) Pacer dependent patients were excluded No abandoned or epicardial leads	1.5T Standard sequences	Cardiac scans • 55 cardiac • 60 brain, C/T/L Spine • 7 thoracic • 3 vascular	Primary: Frequency of adverse events within 3 h after the MRI scan • Requiring life-support procedures • Not requiring life-support procedures • Device modification • Any other adverse event related or unrelated to MRI • Secondary: increase in blood markers and rate of adverse events at follow-up (myoglobin, myocardial band isozyme, troponin)	• No adverse events • No device malfunctions • No significant changes in markers of myocardial necrosis
**Padmanabhan, at al.**[30] 2018 United States	80 patients with abandoned leads 97 scans Rochester, Minnesota, USA	Single center retrospective observational cohort study Mayo Clinic, USA January 2008 to March 2017	Abandoned leads ONLY Non-MR-conditional PPM (n = 31 patients) or ICD (n = 19 patients) or CRT-D (n = 13 patients) or CRT-P (n = 2 patients) or no device (n = 15 patients)) 10 patients with epicardial leads 4 patients with fragmented leads	1.5T Standard sequences	No cardiac scans • 38 head • 22 chest • 29 lumbar • 8 extremity	Primary endpoint: • Difference in adverse event rate and post-MRI serum cTnT value bewteen the study cohort and control group • Death • Generator failure • Lead failure • Loss of capture • Observed atrial arrhythmia • Ventricular arrhythmia • Electrical reset Secondary endpoints Adverse events in the performance of MRI Significant change in device parameters post-MRI cTnT values pre- and post-MRI • Contribution of body part scanned, number of ICD coils, and multiple MRI scans on cTnT values	• No adverse events • No evidence of myocardial injury (cTnT)
**Nyotowidjojo, et al.**[31] 2018 United States	238 patients 339 MRIs Tucson, Arizona, USA	Single center retrospective observational cohort study University of Arizona, USA December 2013To July 2016	Non-MR-conditional PPM (n = 111 patients) or ICD (n = 89 patients) or CRT-P (n = 2 patients) or CRT-D (n = 36 patients) Abandoned leads (n = 6 patients) Epicardial leads (n = 7 patients)	1.5T Standard sequences	Cardiac scans • 73 cardiac • 8 chest • 240 non-thoracic	• Adverse clinical outcomes • Arrhythmias Patient reported symptoms	• 1 full power on reset (patient with a CRT-D device which was reprogrammed successfully) • No adverse CIED complications or clinical outcomes • No significant differences between thoracic and non-thoracic scans
**Vuorinen, et al.**[28] 2019 Finland	793 patients 1000 MRIs Helsinki, Finland	Single center retrospective cohort study University of Helsinki, Finland November 2011 to April 2017	Non-MR-conditional PPM (n = 739 scans); ICD (n = 45 scans); CRT-D (n = 31 scans)’ CRT-P (n = 0 scans) All devices except one were implanted in 2003 or later 22 scans in 17 patients with abandoned leads, including 1 patient with an abandoned epicardial pacing lead	1.5T Standard sequences	Cardiac scans • 144 cardiac • 555 head/spine • 15 thoracic • 200 abdomen/pelvis • 131 extremity/joint • 12 other	• Generator failure • Power-on reset • Clinically relevant changes in pacing threshold or sensing requiring system revision or programming changes • Unexpected battery depletion • Inhibition of pacing • Patient reported events	• 1 pacer dependent patient fell into elective replacement indicator (ERI) mode due to a temporarily programmed high output voltage. • 1 non-pacer dependent patient had the device fall into full electrical reset mode due to electromagnetic interference (later reprogrammed without issues). • 1 patient had a noise reversion notification which was later reprogrammed without issues.
**Gupta, et al.**[26] 2020 United States	532 patients 608 MRIs Falls Church, Virginia, USA	Single center prospective observational cohort study INOVA Heart and Vascular and Virginia Heart, USA September 2015 to June 2019	Non-MR-conditional devices (279 pacemakers; 184 ICDs; 26 CRT-P; 105 CRT-D; 2 subcutaneous ICD; 1 hemodynamic monitor; 25 scans with abandoned leads) • 121 pacemaker dependent patients • 43 ICD and device dependent patients • 14 CRT-D and device dependent patients	1.5T Standard sequences	Cardiac scans • 69 cardiac • 174 head • 221 C/T/L spine • 22 hip/pelvis sacrum • 21 shoulder • 30 knee • 49 abdomen • 22 other	• Lead impedance change • Lead sensing change • Lead threshold change • Battery voltage change • Rhythm changes • Oxygen desaturation • Heart rate changes • Blood pressure changes • Patient symptoms • Syncope • Cardiac arrest • Death	• 1 Patient with transient change in lead impedance with return to baseline • No patient events
**Schaller, et al.**[41] 2021 United States	139 patients 200 MRI scans with at least one abandoned leads Philadelphia, Pennsylvania, USA	Single center retrospective observational cohort study University of Pennsylvania, USA January 2013To June 2020	Active devices with abandoned leads: • 51 single and dual chamber pacers • 81 single and dual chamber ICDs • 61 biventricular pacers/ICDs • 4 subcutaneous ICDs • 3 other devices Abandoned leads • 55 right atrial • 172 right ventricular • 6 coronary sinus • 4 left ventricular • 5 lead fragments • 1 subcutaneous array 64 patients were pacer dependent	1.5T Standard sequences	Cardiac scans • 50 cardiac • 1 chest • 140 face/orbit/ head/brain/neck and C/T/L spine • 15 abdomen/pelvis and rectum • 4 prostate • 9 shoulder, knee, hip, foot, ankle	• Variation in pre- and post-MRI capture threshold of 50% or more, sensing 40% or more, and lead impedance of 30% or more • Burning or pulling sensations in the chest or device pocket • Sustained tachyarrhythmias during MRI • Changes in vital signs attributable to MRI-related programming changes • Power-on resets • Change in pacing rate	• No abnormal vital signs or sustained tachyarrhythmias • No changes in battery voltage, power-on reset events, or changes of pacing rate • Transient decrease in right atrial sensing in 4 patients • Transient decrease in left ventricular R wave amplitude in 1 patient • Sternal heating resolved with premature cessation in 1 patient with an abandoned subcutaneous array
**Bhuva, et al.**[35] 2022 United Kingdom and United States	970 patients 1148 MRIs 615 scans with non-MR conditional systems 111 MRI scans with mismatched CIED-lead vendors; 105 MRI scans with abandoned, epicardial, or very old leads (pre 2001), or scanned < 6 weeks post implant 533 scans with MR conditional systems London, UK and Philadelphia, Pennsylvania, USA	Multi-center prospective (Barts Heart Center, UK and University of Pennsylvania, USA) and retrospective (Royal Brompton Hospital, UK) cohort study 2014 and 2019	Non-MR-conditional PPM (n = 330 scans), ICD (n = 168 scans), CRT-P (n = 26 scans), and CRT-D (n = 91 scans) MR-conditional PPM (n = 332 scans), ICD (n = 149 scans), CRT-P (n = 15 scans), and CRT-D (n = 37 scans) Abandoned leads, permanent epicardial lead,devices manufactured prior to 2001, were included as non-MR conditional scans	1.5T Standard sequences	Cardiac scans Non-MR-conditional devices: • 185 cardiac • 158 spine • 202 head • 91 abdomen/pelvis • 26 extremity/joint • 3 other MR conditional devices • 321 cardiac • 122 spine • 93 head • 46 abdomen/pelvis • 9 extremity/joint • 6 other	• Death, lead failure, sustained symptomatic or life-threatening arrhythmia, complete or partial electricalreset, generator malfunction, inappropriate inhibition of pacing, or inappropriate anti-tachycardia therapies.	• 2 safety events with non-MR conditional devices • 1 scan with an inaccurate battery status fault code requiring generator change (generator was already under a manufacturer advisory) • 1 scan where MRI not performed due to tachycardia on scan initiation • No deaths or lead failure • No complete or partial electrical resets • No inappropriate inhibition of pacing • No inappropriate anti-tachycardia therapies during or immediately after MRI
**Fluschnik, et al.**[34] 2022 Germany	97 patients 132 MRI scans Hamburg, Germany	Single center retrospective cohort study April 2020 to May 2022	Non-conditional devices (n = 35 scans, including 11 scans with pacer dependent patients) Conditional devices (n = 97 scans, including 15 scans with pacer dependent patients)	3T Standard sequences	Cardiac scans Non-MR conditional devices: • 2 cardiac • 2 thoracic • 23 head • 4 abdomen/pelvis • 2 whole spine/aorta • 4 cervical/ lumbar spine MR Conditional devices: • 2 cardiac • 54 head • 16 abdomen/pelvis • 11 whole spine/aorta • 10 cervical/lumbar spine • 2 extremity/joint	• All-cause death • Arrhythmias • Loss of capture • Inappropriate anti-tachycardia therapies • Electrical reset Lead or generator failure during or shortly after MRI	• No adverse events occurred during or shortly after MRI

### 3.2 Previous studies assessing MR safety in patients with mismatched CIED-lead vendors

The CIED system as a whole, even if individual components are classified as MR-conditional, may fall outside of labeling if the patients have mismatched CIED-lead vendors. As shown in Table 1, a combined prospective/retrospective study with 246 generator models, 210 lead models and 638 unique generator-lead combinations published by Bhuva et al. reported no increased risk of MRI in patients with mismatched device-lead vendors compared to those with matched vendors [35]; this study was consistent with a smaller previous study [36]. While these two initial studies are encouraging, it should be noted that they do not cover all potential combinations/permutations of such mismatches.

### 3.3 Previous studies assessing MR safety in patients with abandoned leads

The Center for Medicare and Medicaid Services (CMS) specifically noted the presence of abandoned leads as an exclusion from their policy endorsing reimbursement for MRI studies performed in patients with non-MR-conditional CIEDs, citing a lack of evidence for MR safety in this setting [37]. This was also an exclusion from the recommended protocol in the 2017 HRS consensus statement [17] and has led many institutions to exclude patients with abandoned leads from MRI.

Abandoned or retained permanent leads are disconnected from a pulse generator and may be capped with plastic. Potential risks of imaging patients with abandoned leads include RF-induced heating [38], [39], [40], alteration of capture threshold [41], and discomfort [41], [42]. Several smaller studies published prior to the CMS 2018 policy showed no adverse events in patients after MRI with abandoned leads [30], [44], [45]. Recent studies of 139 patients with 243 abandoned leads undergoing 200 MRIs [41] and of 40 patients with abandoned leads [35] showed no serious safety events, including with epicardial leads which were ∼10% of the sample. However, the authors reported sufficient heating to require MRI cessation in one patient with an abandoned subcutaneous array, emphasizing the need for special care in atypical or under-studied configurations. An accompanying editorial noted that the risk of undergoing MRI in the presence of abandoned leads was likely much lower than the risk of lead extraction prior to MRI [46]. A registry study performed at Mayo Clinic included 80 subjects with non-MR conditional devices undergoing 97 MRI studies with 90 abandoned leads in situ. These patients underwent MRI without evidence of CIED dysfunction, arrhythmias, discomfort during the scan, or biochemical evidence of myocardial injury [30]. Additionally, a recent expert consensus concluded that scans in patients with abandoned leads could be performed using the same safety protocols used for leads connected to generators [47]. Based on the available evidence, some experienced centers with well-integrated multidisciplinary teams have proceeded to image patients with abandoned leads given the higher albeit low incremental increased risk. Considerations for imaging patients with abandoned leads is further discussed in Section V below.

Temporary epicardial pacemaker leads placed at the time of cardiac surgery may be cut at the skin leading to retained fragments. These are generally believed to be unlikely to cause harm during an MRI exams, which can be performed at 1.5T or 3T, and consensus statements have recommended against screening by questionnaire or chest X-ray for retained temporary epicardial leads [47].

### 3.4 Potential device malfunction complications during MRI

Power-on reset switches device programming to ventricular inhibited pacing and, in the setting of ICD systems, re-enables tachyarrhythmia functions. Therefore, a reset does not withhold appropriate brady- or tachy-arrhythmia therapies in the absence of noise; but if scanning continues, pacing may be inhibited, and tachyarrhythmia therapy attempts may be made due to sensing of electromagnetic noise. Thus, a reset must be recognized (often by a subtle change in programmed pacing rate to 60 beats per minute [bpm], or less subtle inhibition of pacing). In the majority of cases, the reset is transient, and programming can be restored with no effects on future device function. However, when permanent reset is observed, the generator must be replaced to allow optimal individualized device programming.

### 3.5 Our recommendations for optimizing MR Safety in patients with CIED

Contemporary recommendations for implementing standardized protocols to optimize MR safety in patients with CIED have been published [19], [20]. These highlight a need for establishing cross-departmental teams with responsible team lead(s) to identify site-specific adaptations to such protocols and to monitor program performance. CIED MR safety protocols are aimed at providing algorithm-driven, stepwise instructions to specific team members during referral, pre-scan, scan, and post-scan periods. The responsible team includes members from the imaging service, cardiology / electrophysiology, as well as referring providers.

**Fig. 1:**
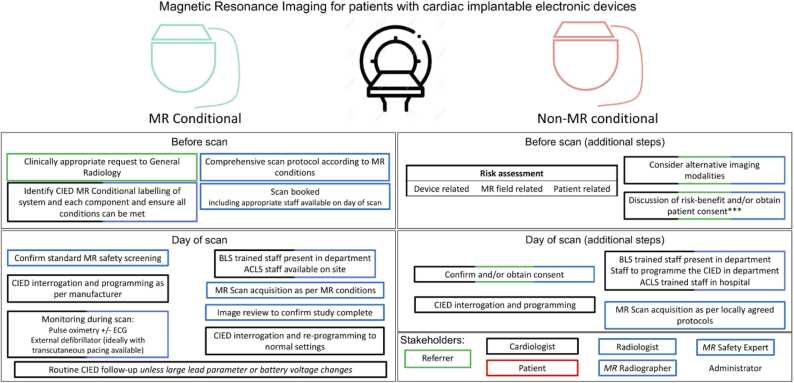
Recommendations for planning and performing MRI scans in patients with CIEDs. *Higher risk scenarios include the presence of epicardial, abandoned leads, fractured; recent implantation; battery at elective replacement indicator/ requires replacement; deactivated systems; lead parameters outside manufacturer recommendations and other implants present. Appropriate person obtaining and confirming consent should be performed as per local protocol. ACLS: adult cardiac life support; BLS: basic life support; CIED: cardiac implantable electronic device; ERI: elective replacement indicator; SAR: specific absorption rate.

Protocol requirements can be broadly organized into planning (prior to day of scan) and procedural (day of scan) tasks, as illustrated in Fig. 1. At time of patient referral, immediate priority is placed on identifying whether the patient has an isolated MR-conditional system (inclusive of generator and leads) that permits entry into manufacturer-recommended pathways for safe MR performance, versus all other patients, who enter a non-MR-conditional pathway (Fig. 1). Regardless of pathway, incremental factors are considered that may influence risk versus benefit estimation. These include the appropriateness of the referral, availability of alternate testing, anticipated location of generator and its influence on diagnostic quality, status of generator battery, and the presence of abandoned or fractured leads. A chest X-ray should be ordered if a recent one is unavailable to determine the presence of abandoned or fractured leads. These and other unique scenarios (such as MR-conditional systems with mismatched components, epicardial or non-standard lead configurations, etc.) are discussed in detail within a recent consensus statement of the Joint British Society [20]. Finally, capacity of the patient to undergo pre-procedural device reprogramming safely must be considered, aimed at identifying pacemaker dependent patients where asynchronous pacing may not be achievable. An appropriate discussion of the relative risk and benefit should then be undertaken with each patient prior to scheduling of CMR, while considering disease specific benefits of CMR relative to alternative imaging modalities.

Content modified with permission from Bhuva et al. [20].

On the day of MR procedure, a coordinated set of tasks are required between the device clinic/electrophysiology and imaging service. CIED device interrogation and programming to MRI mode is first performed, typically to “OVO” or “ODO” mode unless the patient is pacemaker dependent where asynchronous “VOO” or “DOO” modes are recommended. The patient is then transferred to the MR department to undergo a tailored MR protocol with intra-scan monitoring including electrocardiogram (ECG), pulse oximeter, and blood pressure. During the scan, a resuscitation cart and advanced cardiac life support (ACLS) trained personnel should be available within the MR department, and a pacing system analyzer and ACLS trained team present in the hospital. For a non-MRI-conditional CIED, informed patient consent must be obtained prior to the patient entering the MR scanning room following a review of standard MR safety screening for non-device related contraindications. Scanning is recommended to be performed at 1.5T for all non-MR-conditional CIEDs and is preferred over 3T for all MR-conditional devices to mitigate field-related artifacts. Further, evidence supports that patients with left anterior thoracic CIEDs may experience less lead tip heating when imaged in a feet first orientation [48]. All patients should be advised to report discomfort or excessive heating, and rhythm monitored continuously throughout the scan, although special considerations may be necessary in children and other special populations where sedation or anesthesia are frequently required. Optimized CMR protocols are discussed elsewhere in this consensus statement; however, it is advised that all images be reviewed by the imaging clinician prior to study completion to ensure diagnostic quality and avoid repeat testing. Adherence to a peak whole-body specific absorption rate (SAR) below 2.0 W/Kg has in general been advised. It is advisable to stay well below the 2.0 W/kg SAR limit, to account for variations in SAR calculation by the various MR system vendors. Alternatively, B1+rms is a vendor neutral measurement and may be a better metric for estimating safety uniformly across all vendors. However, a recent analysis of 2028 MR examinations without SAR restriction failed to identify any associations between SAR, db/dt, scan duration and changes in CIED parameters immediately following MRI [49]. To assess for such changes, repeat CIED interrogation is mandatory for all patients immediately following the MR examination with any significant changes in device or lead parameters reviewed by an electrophysiologist. Regarding the definition of significant device parameter changes, a set of pre-defined, conservative thresholds for significant changes attributable to MRI (outside the range of normal measurement fluctuation) were developed when designing prospective studies for conditional devices (a decrease in sensed P wave amplitude ≥ 50%; a decrease in sensed R wave amplitude ≥ 25%; an increase in capture threshold ≥ 0.5 volts (V); an absolute change in pacing lead impedance ≥ 50 Ω; an absolute change in high-voltage lead impedance ≥ 3 Ω; a decrease in battery voltage ≥ 0.04 V) [12], [50]. The patient then returns to their routine CIED interrogation and surveillance plan.

## 4 Physics of MR Safety

In general, radio-frequency (RF)-induced lead-tip heating and gradient magnetic field induced current induction are the principal safety concern for most CIEDs. Even with non-MR-conditional systems, clinical MRI protocols and in vivo measurements yield temperature changes < 0.5 °C, and the extent of heating and risk of tissue damage is minimal if safety protocols are followed [7]. Additionally, with conventional implant conditions, the amplitude of low frequency induced current is < 0.5 mA and unlikely to result in myocardial capture [51]. Patients with Food and Drug Administration (FDA) approved MR-conditional devices can safely undergo an MRI exam with the protocol adhering to the conditions for the implanted device, which frequently requires limited SAR or B_1_+rms, defined as the average effective RF magnetic field generated by the RF transmit coil for a given pulse sequence. Note too, that patients with implanted CIEDs may need to undergo MRI of any body part depending on the clinical indication for the exam [52]. It is also important to note whether a device is MR-conditional for 3T or 1.5T or both. It is wrong to assume that a device approved at 3T will necessarily be safe at 1.5T (or any lower static magnetic field [B_0_] field) MRI systems with a range of B_0_ fields, gradient performance, and RF transmit specifications continue to be marketed. Therefore, it will be important to remain vigilant about the appropriateness of obtaining an MRI exam for a given combination of the CIED’s conditional labeling and the MRI system used for the exam. For more technical details on physics of MR safety, see Appendix I.

## 5 Legal-Risk Management Considerations

Patients with CIEDs have the same clinical indications to undergo CMR as those without devices. However, the presence of the CIED requires an assessment of patient specific risks in the MR environment relative to the disease specific diagnostic benefits of CMR. Risks and benefits of diagnostic strategies and therapeutic treatments are managed by care providers as part of routine clinical care. This allows for discretion informed by shared decision making in the context of disease severity and available medical therapies or procedures. Management decisions should consider the risk of a negative event due to the underlying disease relative to the potential benefits from CMR.

CMR of patients with CIEDs has additional risks associated with an active device with leads terminating at the myocardium. While the risks in the MR environment are minimized given certain imaging conditions, in rare instances an adverse event can still occur. Patients with CIEDs undergoing MRI can be grouped into the following risk categories (see Fig. 1 for cross-reference):

(1) MR-conditional CIED systems (generator and leads) approved for use in the MR environment.
(2) Non-MR-conditional CIED systems without intracardiac abandoned/fractured or surgically placed permanent epicardial leads.
  a. MR-conditional CIEDs but utilizing intracardiac leads falling outside of the conditional requirements.
  b. Non-MR-conditional CIED generators.

**Fig. 2:**
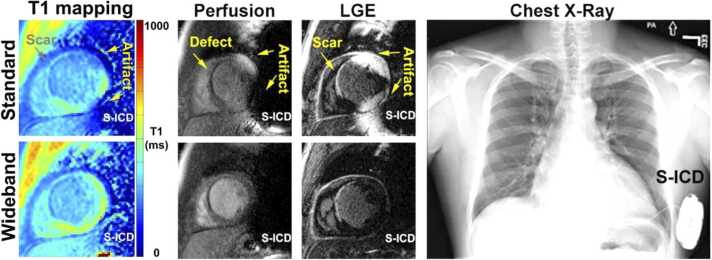
(Top row) Conventional T1 mapping, perfusion, and LGE of a patient with an S-ICD (see right panel) shows image artifacts, whereas (bottom row) the corresponding wideband pulse sequences suppressed image artifacts. S-ICD: subcutaneous ICD.

(3) Patients with any CIED who also have
  a. Abandoned or fractured (ungrounded) leads terminating in the heart.
  b. Epicardial (surgically placed) permanent leads.

Patients in category 1 can safely undergo CMR performed according to the conditional labeling of the CIED system. If CMR can be performed according to the conditional labeling, such scans are on-label and considered standard of care procedures.

Patients in category 2 fall into the national coverage determination (NCD) for CMS reimbursement for beneficiaries based on the available evidence. For payment CMS requires the following stipulations: (1) imaging performed at 1.5T, (2) benefits and harms communicated to the patient or the patient’s delegated decision maker, (3) the CIED is programmed appropriately before the MR scan, (4) a physician, nurse practitioner, or physician’s assistant with CIED expertise directly supervises the patient during the scan, (5) patients are observed visually and with voice communication, with equipment to assess vitals and cardiac rhythm, (6) a practitioner with advanced cardiac life support (ACLS) training is present for the duration of the scan, and (7) the device is interrogated immediately after the MRI to detect and correct any abnormalities resulting from the scan. Category 2 patients are higher risk but for a clinically indicated scan the risks are small and manageable relative to the benefit of clinically actionable information obtained from CMR.

Patients in category 3 fall outside of the CMS NCD, as the review determined that there was insufficient evidence to support the safe scanning of such patients. CMS believes these patients fall into highest risk category, although objective evidence of potential more harm than the other two categories is lacking [41], [42]. Although scanning such patients has been performed safely, these are best suited to experienced centers with well-established programs relying on close collaboration between radiologists or non-invasive cardiologists, MR technologists, and electrophysiologists. Looking forward, establishing a dedicated CIED registry may better align risk and potential benefit in category 3 patients. Additionally, the lack of reimbursement for Medicare beneficiaries reduces enthusiasm for CMR of category 3 patients at many centers.

Despite established protocols and local expertise, an adverse event, while exceedingly rare, can still occur in any patient category. In such situations the patient's care will be primary with a decision to proceed or not based on their status and best clinical interests. Potential scenarios where CMR may pose greater risk: (a) patients who are unable to respond to painful stimuli innately have one less margin of safety – this includes patients who are sedated. Additional precautions during setup and scanning may be beneficial to consider; (b) legacy non-MR-conditional CIED generators manufactured before 2000 may behave erratically in the MR environment, but are exceedingly unlikely to be encountered in current clinical practice. Any currently implanted and functional generator is likely to have sufficient filtering to proceed with MRI provided that safety protocols are followed. Leads implanted prior to 2000, however, remain abundant in practice and can be considered as category 2 systems as long as their function remains normal. Nevertheless, noting the date of implant is recommended prior to considering a patient with a non-MR-conditional CIED to be in category 2.

In summary, the risks during an MRI examination include those related to the underlying disease with the addition of MRI without a CIED, MRI plus MR-conditional CIED, or MRI plus non-MR-conditional CIED. Discussing the relative risks and the clinical response required should an adverse event occur around the time of the MRI allows the patient to make an informed deduction to proceed in risk categories 2 and 3. MRI of patients in risk category 1 is considered on-label provided that MRI follows the conditional guidance of the manufacturer. For categories 2 and 3, the imaging center should collaborate with the local legal/risk management team to establish a consistent patient consent procedure, through which shared decision making can be accomplished documenting informed consent. For suggested informed consent statements, see Table 2. Additionally, standard documentation of the procedures for MRI of CIED patients should to be included in the CMR report. Example wording is provided in Table 3.

**Table 2:** Suggested statements to use when describing risk during consent for patients with different functioning non-MR-conditional cardiac implantable electronic devices (CIED). Content modified with permission from Bhuva et al. [20]. These statements should be used in addition to discussing the MRI procedure, potential benefits and alternatives. This list is intended for common scenarios, and not as an exhaustive list. * ‘Mismatched’ CIEDs have MR-conditional generators and non-MR-conditional leads; or MR-conditional components from different manufacturers.

MRI Scanning Scenarios with Different CIEDs and Leads	Recommended risk statement to discuss with the patient. *The MRI procedure, benefits and alternatives should also be discussed with the patient with the opportunity for them to have additional queries addressed by an appropriate clinician.*
**Intermediate and Higher risk scenarios (formal written consent required)**	
**Non-MR-conditional CIED (No additional higher-risk scenarios)**	You have been referred for a magnetic resonance imaging (MRI) scan. Your pacemaker/ defibrillator has not been formally approved by the manufacturer to undergo MRI scanning. After discussing the possible benefits, risks, and alternatives with your referring doctor, the decision to perform the MRI scan has been made. Serious complications related to MRI occur in < 1 in 2000 patients (∼0.05%) with these devices overall. These include, but are not limited to: - - Cardiac device damage - - Irregular/ abnormal heart rhythms - - Excessive tissue heating Emergency or urgent cardiac device replacement may be needed and will be performed if required.
**Additional Intermediate and Higher risk scenarios (formal written consent required)**	
**Non-MR-conditional CIED generators implanted before 2005**	[in addition to above] Due to your device’s age, the risk may be slightly higher – with ∼2% risk of (generally temporary) program changes to “factory settings”.
**Non-MR-conditional CIEDs implanted before 2000**	[in addition to above] There is less evidence for scanning patients with old devices implanted before the year 2000. We know that the older devices are more sensitive to MRI and therefore the risk is likely to be higher.
**Abandoned lead(s)**	[in addition to above] Having a pacemaker or defibrillator lead which is not attached to a generator may result in heating at the lead tip in your heart, which could theoretically cause tissue damage. To date, there have been no reported problems in patients being scanned with these leads, although the number of these patients is relatively small. We would ask that you inform staff immediately if you feel any discomfort.

**Table 3:** Suggested documentation in CMR reports for CIED patients.

Category	Technical note
1	Due to the patient's implanted MR conditional pacemaker/ICD, scanning was performed in Normal Operating Mode. Cardiology personnel programmed the device appropriately before and after the MRI and monitored the patient throughout. No immediately apparent complications.
2	Due to the patient's non-MR-conditional cardiac implantable electronic device, written informed consent was obtained prior to exam. Scanning was performed in Normal Operating mode. Cardiology personnel programmed the device appropriately before and after the MRI and monitored the patient throughout. No immediately apparent complications.
3	Due to the patient's non-MR-conditional cardiac implantable electronic device with [fractured leads(s), abandoned lead(s), epicardial lead(s)], written informed consent was obtained prior to exam. Scanning was performed in Normal Operating mode. The predicted whole-body SAR did not exceed 2.0 W/kg. Cardiology personnel monitored the patient throughout. No immediately apparent complications.

## 6 Pulse sequence and MRI protocol optimization

We recommend that healthcare providers carefully evaluate the benefit of CMR in CIED patients, because unoptimized CMR protocols are likely to yield suboptimal or even non-diagnostic images, and even optimized CMR protocols may yield suboptimal or even non-diagnostic images in a particular combination of device, generator placement, and patient body habitus (e.g., subcutaneous ICD [S-ICD] of a thin patient).

### 6.1 Origin of image artifacts in CIED patients

There are several reasons why CMR images may be degraded in patients with a CIED. First, the CIED pulse generator, which contains a battery, circuitry, reed switches, and a titanium can, causes significant macroscopic field variations. The B_0_ center frequency may be shifted on the order of kHz. As a reference, B_0_ variation across the heart at 1.5T in the absence of CIED is approximately 70–100 Hz [53]. Image artifacts induced by a CIED include signal voids from dephasing, image distortion from off-resonance, and hyperintense signals in regions where preparation RF pulses are not excited due to large center frequency shift. For these reasons, pulse sequences that are particularly sensitive to off-resonance, such as balanced steady state free precession (b-SSFP), should be avoided for CMR in CIED patients. Another reason why b-SSFP pulse sequences should be avoided is that they typically use larger flip angles, which deposits high RF energy to the patient and CIED (i.e., safety concern). Instead, gradient recalled echo (GRE) pulse sequences should be used in CIED patients. Disadvantages of GRE pulse sequences compared with b-SSFP include lower blood-to-myocardium contrast and higher degree of flow-inducted signal voids. Second, the intracardiac leads (wires) cause benign field variations, typically leading to small signal voids around the wires. Third, CIED patients often have a higher burden of heart disease and arrhythmia than matched patients with no CIED. Arrhythmia and poor ECG tracing are a major source of image artifacts for “segmented k-space” pulses sequences that acquire data over multiple heartbeats with ECG synchronization. Fourth, CIED patients often have a higher burden of dyspnea, which is a source of image artifacts for breath-hold pulse sequences. The following section will describe techniques for mitigating such image artifacts.

### 6.2 Techniques for mitigating image artifacts in CIED patients

Multiple methods can be used to mitigate image artifacts caused by CIED. Signal voids due to dephasing usually occur around the device pulse generator, which is typically located 5–15 cm away from the heart (if implanted below the left clavicle). Depending on the distance from the generator to the heart and the material used by the generator, these signal voids may or may not affect the heart. Both location and size of signal voids depend on device type and implantation location. Prescribing smaller voxel size (i.e., thinner slice) or minimizing the echo-time (TE)(e.g., shorter RF pulse, high receiver bandwidth, partial echo) during CMR can mitigate this challenge to some degree. Another simple strategy to mitigate image artifacts for patients with left-sided CIED implant is raising the ipsilateral arm during the scan, which physically increases the distance between the heart and CIED; for patient comfort, it may be possible to stabilize the raised arm with gauze bandage or elastic band [54]. For patients with right-sided CIED implant, it may be possible to use standard CMR pulse sequences without significant image artifacts on the heart. Device-dependent B_0_ off-resonance also causes geometric distortions. In conventional CMR with Cartesian k-space sampling, these distortions occur in the frequency-encoding direction as well as the slice/slab direction. During a frequency-encoding readout, regions with off-resonance accumulate additional signal phase, which, during the Fourier imaging process, is encoded to a different location in the frequency-encoding direction. For example, with a 2 kHz off-resonance and a readout bandwidth of 1000 Hz/pixel, the distortion would be 2 pixels. Therefore, frequency-encoding distortion can be effectively reduced by using a larger readout bandwidth. Distortion in the slice/slab direction is due to a different mechanism. Large off-resonance distribution in the slice direction can result in a distorted 2D slice being excited when the excitation pulse is played; rather than exciting a 2D plane, a curved 2D slice may be excited. Consequently, anatomy outside of the prescribed imaging plane can be erroneously encoded to the intended slice. If the curved 2D slice traverse through a signal void area outside of the intended slice, the signal void will also be present in the image. These slice distortions can be effectively mitigated using multi-spectral methods [55], albeit with prolonged scan time.

The large device-dependent B_0_ off-resonance can cause an additional type of artifact for CMR pulse sequences with preparation modules such as inversion recovery (IR) or saturation recovery (SR). The spectral bandwidth of these preparation pulses is typically on the order of 1–2 kHz, whereas it is about 5–6 kHz for a typical excitation pulse used in a GRE pulse sequence. The off-resonance caused by the CIED are typically outside of the spectral bandwidth of the IR or SR pulses, but within the bandwidth of excitation pulses. Therefore, pulse sequences such as LGE, perfusion, and CMR relaxometry are vulnerable to image artifact caused by insufficient magnetization preparation due to limited spectral bandwidth of the preparation pulses. A wideband technique, initially proposed by Rashid et al. [56] for IR LGE, has been adopted for T1 mapping [57], [58] and perfusion [59] CMR. The preparation module is modified to enable a wider spectral bandwidth, e.g. 3.8 kHz IR pulse used by Rashid et al. [56] and 9.2 kHz SR pulse by Hong et al. [59], such that the off-resonant magnetization is effectively rotated by the prescribed flip angle of the preparation module. This family of wideband CMR pulse sequences have been demonstrated to be effective in removing these image artifacts in clinical practice [32], [60], [61], [62]. An example shown in Fig. 2 demonstrates the use of wideband IR and SR pulses for improved T1 mapping, perfusion, and LGE CMR in a patient with an S-ICD compared with the corresponding standard pulse sequences.

### 6.3 Pulse sequence recommendations

Table 4 summarizes imaging parameters for cine, phase-contrast, T1 mapping, T2 mapping, LGE, and perfusion pulse sequences for scanning CIED patients. Imaging centers with local expertise in MR physics should modify their imaging protocols adhering to these recommendations. As of to date, there are no “wideband” T2* pulse sequences specifically designed for CIED patients. T2* measurements are unlikely to be reliable due to large B0 variations across the heart caused by the pulse generator, particularly in patients with implantable defibrillators (e.g., ICD, CRT-D). For patients with thalassemia implanted with pacemakers that are distal to the heart, in whom myocardial T2* measurement is clinically relevant for monitoring chelation therapy, it may be possible to perform serial imaging with both magnitude (T2* ) and phase (B0) reconstructions to measure changes in T2* over time in regions where B0 variation is not severe, as identified by the B0 map. In the absence of robust evidence (e.g., T2* versus myocardial biopsy), the radiologists or non-invasive cardiologists must interpret T2* measurements from CIED patients with caution. Alternatively, the imaging facility may consider wideband T1 or T2 mapping pulse sequences, because they are less sensitive to CIEDs than T2* mapping. However, the disadvantage of T1 and T2 mapping is that there is less historical evidence for their utility for monitoring chelation therapy.

**Table 4:** A summary of different CMR pulse sequences and their recommended settings. All pulse sequences should use spoiled gradient echo readout. *SR: saturation recovery; IR: inversion recovery.*

Pulse Sequence Type	Key technical considerations
Cine	Short RF pulse (<1 ms) with low flip angle (10-15°), receiver bandwidth > 500 Hz/pixel; if severe arrhythmia, consider real-time cine
Phase-contrast	Short RF pulse (<1 ms) with low flip angle (10-15°), receiver bandwidth > 500 Hz/pixel; if severe arrhythmia, consider real-time cine
T1 mapping	Wideband SR or IR preparation pulse, short RF pulse (<1 ms) with low flip angle (10-15°), receiver bandwidth > 500 Hz/pixel;
T2 mapping	Wideband T2-preparation pulse, short RF pulse (<1 ms) with low flip angle (10-15°), receiver bandwidth > 500 Hz/pixel;
LGE	Wideband IR preparation pulse, short RF pulse (<1 ms) with low flip angle (10-15°), receiver bandwidth > 500 Hz/pixel;
Perfusion	Wideband SR preparation pulse, short RF pulse (<1 ms) with low flip angle (10-15°), receiver bandwidth > 500 Hz/pixel;

For centers lacking requisite MR physics expertise, they should work with their vendors’ solutions for scanning CIED patients. For centers lacking access to customized and/or vendor wideband pulse sequences, it may be possible to proceed with non-CIED specific product pulse sequences, albeit at lower diagnostic yield. Table 5 summarizes latest MRI vendors’ solutions for CMR of CIED patients. Imaging centers should consult with their vendors to utilize pulse sequences tailored for CIED patients. It should be noted that conventional commercial product pulse sequences were not designed and FDA-approved specifically for CIED patients. For example, for patients with MR-conditional CIEDs, conducting standard product CMR pulse sequences with b-SSFP readouts (cine, mapping, certain versions of LGE and perfusion) would generate higher SAR (i.e., less safe) than works-in-progress (WIP) pulse sequences with GRE readouts. In this scenario, commercial pulse sequences would be less safe than WIP sequences, even though FDA approval is nominally ascribed for product pulse sequences. In another scenario, for patients with non-MR-conditional CIEDs, any CMR is off-label, so in this context the distinction between product and WIP pulse sequences in terms of regulatory consideration is less meaningful.

**Table 5:** Summary of vendors’ solutions for CMR of CIED patients. IR: inversion recovery; SR: saturation recovery; SAR: specific absorption rate; WIP: work in progress; CIED: cardiac implantable electronic device. *corresponds to pre-release beta versions.

Vendors	LGE	Cine	Phase contrast	T2 mapping	T1 mapping	Perfusion	Additional safe limits
**GE**	Wideband IR (4 kHz) 2D and 3D LGE with spoiled gradient echo, optional free-breathing and AIR Recon DL	2D and 3D spoiled gradient echo	2D and 3D gradient echo	No special protocols available for CIED patients	Wideband IR (4 kHz) 2D spoiled gradient echo with optional AIR Recon DL and motion correction	Wideband SR (5 kHz) 2D spoiled gradient echo with optional AIR Recon DL and motion correction	B1 + amplitude, whole body SAR, head SARand scan duration
**Siemens**	Wideband IR (6 KHz) with HeartFreeze for 1.5T. WIP* for other field strengths	2D spoiled gradient-echo with optional CS acceleration	2D/4D flow with spoiled gradient-echo	No special protocols available for CIED patients	SASHA* with wideband saturation (4 kHz) and spoiled gradient-echo readout	Wideband SR (4 kHz) with 2D spoiled gradient-echo readout	“Implant Suite” WIP* for restricting B1 + rms, body SAR, head SAR
**Philips**	Wideband IR (4 KHz) for 2D and 3D LGE WIP*	2D gradient echo protocols available	2D gradient echo protocols available	No special protocols available for CIED patients	No special protocols available for CIED patients	No special protocols available for CIED patients	“ScanWise Implant” restricts, Whole Body SAR, Head SAR, Gradient strength and slew rate
**Canon**	Actively investigating Wideband IR solutions for 2D and 3D LGE	2D spoiled gradient echo protocols available	2D spoiled gradient echo protocols available	No special protocols available for CIED patients	No special protocols available for CIED patients	No special protocols available for CIED patients	Adaptative model for SAR control

### 6.4 Technologist’s Guide for CMR of Patients with a CIED

There are many considerations a technologist must bear in mind when scanning patients with CIEDs. Patients with CIEDs, in general, have weaker ECG signals than patients with no CIEDs. It is important to use a variety of techniques to get the best ECG signal possible. First, discuss with the monitoring clinical personnel that the technologist needs to place his/her ECG electrodes in the most optimal areas of the chest according to scanner manufacturer recommendations. Occasionally, the ECG signal can be disrupted as the patient is shifted to the scanner isocenter at the beginning of the exam. If this happens, it may be helpful to “relearn” the ECG signal once the patient is at isocenter. It is also possible to notice distortion in the ECG signal, which result in mis-triggering due to the time-varying gradient magnetic fields. Additionally, if the technologist notices ECG disruptions during breath holds, it may be worth doing an ECG “relearn” during a breath-hold.

When beginning the acquisitions, it is important to mitigate the susceptibility artifacts with the available tools, e.g., by using GRE-based pulse sequences. The type, location, and position of the device will all contribute to the size and location of the artifact. For example, an S-ICD on the left side of the chest will be very challenging to image. If the patient has a left-sided device and they are able, consider raising their left arm over their head to move the device a few millimeters further from the heart. Sometimes, even with advanced sequences, it is not possible to remove the artifact from the entirety of the heart. It is important the scanning technologist maintain communication with the radiologist/cardiologist that will be reading the study to determine if further imaging is needed for a given patient, instead of repeating sequences with no improvement in results. Depending on the clinical question, having artifact in part of the heart can still result in a diagnostic exam.

It is important to have a designated person in charge of protocol management that will build the appropriate sequences and parameters to have the lowest SAR possible and maintain the protocols as changes are implemented. It is imperative to remain in normal operating mode during these scans.

Safety is always a very important consideration in MRI, but it is especially important for device patients. For technologists who have been trained over their careers that CIEDs were absolute contraindications for MRI, the thought shift to scanning these patients safely is large. It is important that technologists are thoroughly trained in a facility’s policies and procedures as well as MR safety concepts as it relates to CIEDs to ensure their comfort in caring for and imaging these patients.

## 7 Non-electrophysiologic indications for CMR

Non-electrophysiologic related clinical indications for CMR in patients with CIED include assessment of both ischemic and non-ischemic cardiomyopathies, evaluation of new onset heart failure symptoms and infiltrative diseases, and vascular imaging.

### 7.1 Cardiomyopathy

For evaluation of cardiomyopathy, key sequences include cine for quantification of cardiac chamber size, function and strain; LGE for evaluation of replacement fibrosis and expansion of the extra-cellular space; T2 mapping for evaluation of edema and inflammation; and T1 mapping and extracellular volume (ECV) for evaluation of interstitial fibrosis and infiltration.

Multi-plane LGE imaging is a key sequence in the CMR protocol for evaluation of known or suspected cardiomyopathy, including in patients with CIEDs. However, artifact is relatively frequent with standard techniques. Wideband LGE sequences are useful to suppress image artifact induced by the generator of a CIED [56], [63]. Wideband segmented breath-hold and wideband single-shot (SS) free-breathing LGE pulse sequences have both been shown to result in improved image quality compared to standard LGE [61].

Assessment of myocardial T1 values using parametric mapping techniques are increasingly being integrated in clinical protocols for assessment of cardiomyopathy resulting in higher diagnostic confidence and accuracy [64]. Parametric mapping allows for non-invasive quantitative myocardial tissue characterization. Native T1 mapping provides unique insight into patients with interstitial fibrosis and infiltrative disease, including cardiac amyloidosis and Fabry disease [65], [66], [67]. However, accuracy may be reduced in patients with CIEDs due to image artifacts. Wideband T1 mapping using broadband saturation [57] or inversion [58] pulse with GRE readout has been shown to suppress image artifacts and relatively accurate T1 measurements; however, they need to be evaluated further in CIED patients. T2 mapping is also susceptible to image artifacts caused by the device. A wideband T2 preparation pulse combined with GRE readout has been shown to reduce image artifact [68]; however, the clinical utility of such imaging remains to be evaluated in patients with CIEDs. While it may be possible to achieve diagnostically useful images in S-ICD patients using wideband LGE [69], further evaluation is warranted [70]. Finally, it should be noted that local reference values obtained using non-wideband pulse sequences from patients with no CIED may not be applicable defining normal values for CIED patients using wideband pulse sequences.

### 7.2 Onset of new HF symptoms in patients with a CIED

**Fig. 3:**
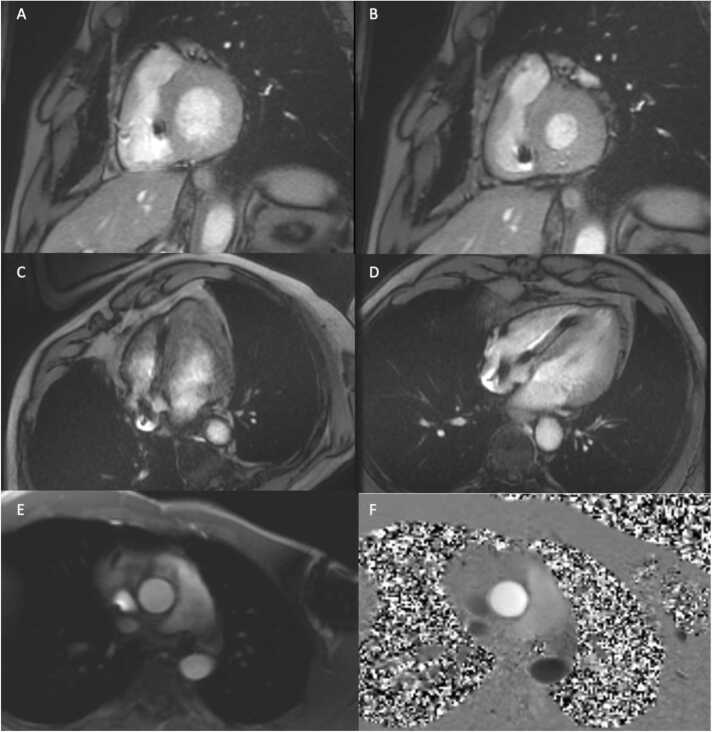
Exemplary CMR images in a patient with a left-sided CIED including short-axis GRE post-contrast images at end-diastole (A) and end-systole (B); 4-chamber GRE images pre-contrast (C) and post-contrast (D); and 2D phase contrast imaging at the ascending aorta (E, magnitude; and F, phase).
• *Valvular Evaluation*
  CMR is the standard of reference in quantifying the extent and severity of valvular heart disease [74]. Although 2D phase contrast imaging with phase encoding in two directions is considered the reference standard, 2D phase contrast imaging with tri-directional encoding and now 4D approaches are in common use removing the impact of plane angulation on accuracy [75]. The pulse sequences used in patients with CIEDs are the same; phase contrast imaging is GRE based and as such relatively insensitive to local field effects. However, quantification near cardiac devices may be impacted and, as standard phase contrast techniques are segmented, image quality is degraded in patients with arrhythmia and dyspnea. The location of the generator may impact aortic root, mid ascending aortic, distal pulmonary, and branch pulmonary measurements. However, measurements at the level of the cardiac valves are not usually affected. Transvenous leads traversing the tricuspid valve plane will cause challenges in direct measurements of tricuspid inflow and assessing the peak velocity. However, the degree of tricuspid regurgitation can be derived from the indirect method, comparing the right ventricular stroke volume with the pulmonic valve forward flow. Attention to internal consistency between the degree of valvular regurgitation and relevant chamber stroke volume is recommended to increase confidence in quantitation of valvular heart disease in patients with CIEDs. Velocity encoding gradient selection and plane positioning is similar to scanning patients without cardiac devices. The degree of flow across shunts, anomalous pulmonary veins, and other connections can be quantified provided the generator or lead artifact does not lead to signal loss at the region of interest [76].

• *Functional Evaluation*
  Although other imaging modalities including echocardiography are able to determine biventricular systolic function and measure chamber size, CMR is considered the reference standard modality [71]. Traversing leads into the RV do not pose significant artifact in contouring the chamber or identifying the tricuspid base plane. Irregular heart rhythms are challenging, as available product GRE pulse sequences require segmented acquisitions [72]. However, end-diastolic volumes can be accurately measured even with a segmented approach, as shown in Fig. 3. Caution should be applied in relying on end-systolic volumes and identifying regional wall motion abnormalities with segmented acquisitions in arrhythmia. Real-time cine CMR techniques allow for detection of regional wall motion abnormalities, though quantification of biventricular size and systolic function is challenged by lower temporal resolution, image artifacts from the generator, and limited spatial resolution [73]. However, new regional wall motion abnormalities and dyssynchrony can be confidently identified with real-time cine CMR methods.

### 7.3 Ischemia imaging in patients with CIED

Patients with CIED frequently develop new symptoms of chest pain or shortness of breath warranting evaluation of ischemia due to suspected coronary artery disease. Although dobutamine stress CMR (DSMR) wall motion assessment is a validated technique for assessing ischemia in other populations, most CIED patients will not be appropriate for DSMR due to inability to achieve target heart rate, tachyarrhythmias that may be exacerbated or precipitated by high-dose dobutamine, and/or underlying left ventricular dysfunction and LV dyssynchrony secondary to RV pacing that may complicate the interpretation wall motion abnormalities at peak stress. Therefore, vasodilator stress perfusion is the preferred method for evaluating ischemia by CMR in patients with CIED.

In non-CIED populations, vasodilator stress CMR perfusion imaging is an established method for evaluation of ischemia characterized by high diagnostic accuracy when compared to coronary angiography and especially invasive fractional flow reserve [77], effective risk stratification for cardiac events by the presence and extent of ischemia [78], and the ability to combine stress perfusion with other CMR imaging techniques including parametric mapping and LGE imaging for a comprehensive cardiovascular exam. As such, stress CMR has received Class I indications for the evaluation of suspected coronary artery disease from the most recent European and U.S. guidelines [77], [79].

In general, device management for vasodilator stress will be similar to the guidelines described elsewhere in this document. However, one unique aspect requiring consideration is the effect of vasodilator medications on heart rate and atrioventricular node conduction. Many CIED patients will have underlying atrioventricular (AV) block which could be worsened by adenosine infusion. In a study of patients with preserved AV conduction but evidence of intermittent AV block on PPM interrogation, a 3-minute test infusion of adenosine led to worsening of AV conduction and a fall in heart rate in 33% of patients [80]. Programming the device to asynchronous pacing in “VOO” or “DOO” mode will prevent bradycardia in susceptible patients. However, patients without significant sinus node dysfunction or AV nodal disease will typically experience an increase in heart rate with adenosine and should have pacing deactivated (“ODO” mode). Because CIED inhibited mode must be turned off to avoid inappropriate inhibition by sensing of electromagnetic impulses from the scanner, an adenosine induced increase in the sinus rate to above the pacing rate will result in competitive pacing – which may be uncomfortable and raises the theoretical possibility of a malignant ventricular arrhythmia precipitated by a pacemaker impulse falling in the vulnerable period of ventricular repolarization (R-on-T phenomenon).

Several single center retrospective studies have reported on the safety of vasodilator stress CMR perfusion in CIED patients (Table 6). The overwhelming majority of the patients included in these studies had MR-conditional PPM or ICD devices. The aforementioned study used an individualized algorithm to decide the appropriate pacing mode based on presenting rhythm and a test adenosine infusion outside of the CMR scanner room. Other studies did not use a test adenosine infusion, basing the decision to pace asynchronously on resting heart rate < 45 bpm [14] or > 1% pacing requirement on device interrogation [81]. No adverse events related to adenosine infusion occurred in any of the studies, and notably no episodes of competitive pacing were reported. Additionally, no changes were seen in pacing capture thresholds, sensing amplitudes, lead impedance, or battery voltage.

**Table 6:** Retrospective studies reporting on the safety vasodilator stress CMR perfusion in CIED patients.

Study	Dates	N	Device type [Field Strength]	Adverse events	CIED changes	Key Findings
Klein-Wiele et al.[14]	3/2014-4/2015	24	MR-conditional PPM	None	None	Safety of adenosine stress CMR
Klein-Wiele et al.[80]	4/2015-12/2016	47	MR-conditional PPM	None	None	Safety of tailored PPM programming scheme for adenosine stress CMR
Pezel et al.[83]	Before 10/2021	224	MR-conditional PPM [1.5T]	None	None	Diagnostic quality in 99%, PCI performed in 33/35 (94%) CMR guided ICA referrals, ischemia and LGE were independent predictors of MACE
Pavon et al.[81]	8/2013-3/2021	66	MR-conditional PPM (N = 36), ICD (N = 28), SQ-ICD (N = 2) [1.5T]	None	None	Diagnostic quality in 98%, non-diagnostic quality in patients with SQ-ICD, critical coronary stenoses 6/6 patients with ischemia referred for ICA
Miller et al.[82]	5/2018-9/2021	20	MR-conditional PPM (N = 10) [all at 3T] MR-conditional ICD (N = 8) [2 1.5T, 6 3T] Non-MR-conditional ICD (N = 2) [4 at 1.5T; 16 at 3T]	None	None	Diagnostic quality in 16/18 (89%) for MR conditional, 0/2 (0%) for MR nonconditional.

Diagnostic image quality was achieved in the majority of patients with MR-conditional devices (80%−90%). The only study to include non-MR-conditional devices reported, in the two patients with non-MR-conditional ICDs, perfusion images were marred by significant artifacts rendering the studies nondiagnostic. Therefore, patients with non-MR-conditional ICDs were subsequently excluded from undergoing stress CMR [82]. The use of newer wideband perfusion pulse sequences significantly reduces artifact level, improves overall visual scores, and even enables quantification of myocardial blood flow (in mL/min/g) [59].

In the limited number of patients who were referred for coronary angiography based on CMR findings, a high percentage were found to have severe coronary stenoses. A report of 224 patients with MR-conditional PPM undergoing adenosine stress CMR suggests that the prognostic ability of stress CMR is maintained in patients with CIED. The rate of cardiovascular mortality and nonfatal myocardial infarction was low in patients without ischemia (0.9%/yr), while the major adverse cardiac event (MACE) rate increased progressively in those with LGE, ischemia, or both LGE and ischemia [83].

### 7.4 Infiltrative cardiomyopathies

Many individuals with infiltrative cardiomyopathies such as cardiac sarcoidosis and cardiac amyloidosis present with high degree heart block or malignant ventricular tachycardia [84] often requiring treatment with a CIED prior to the determination of a specific etiology of their cardiomyopathy. Because of the important role CMR plays in the assessment of infiltrative cardiomyopathies, these individuals are often referred for CMR after CIED implantation. LGE imaging and T1-mapping play a crucial role in the diagnosis of infiltrative cardiomyopathies [85]. Although the diagnostic performance of these two techniques for diagnosing infiltrative heart diseases has not specifically been tested in patients with a CIED, use of the wideband technique effectively suppresses imaging artifact [4], [56], [60], [86] and it is unlikely that the diagnostic ability of LGE imaging and T1-mapping would be significantly diminished in patients with CIED. An important complication of infiltrative cardiomyopathies such as cardiac sarcoidosis is the development of recurrent VT, and CMR LGE imaging can play an important role in predicting freedom from VT following an ablation procedure [87]. Another important role of CMR in patients with infiltrative heart disease is to monitor treatment response. Although not specifically tested in patients with CIED, the change in ECV following therapies for cardiac amyloidosis is increasingly being used to determine the effectiveness of therapies [88]; further evaluation of wideband T1-mapping techniques [57], [58] in CIED patients is warranted. Similarly T2-mapping techniques are increasingly being used to monitor for improvement in active myocardial inflammation following the initiation of immunosuppressive therapy in patients with cardiac sarcoidosis [89]; further evaluation of wideband T2 mapping [68] in CIED patients is warranted.

### 7.5 Other secondary non-electrophysiologic indications

Other secondary non-electrophysiologic indications for CMR in patients with CIEDs include vascular imaging (e.g., for assessment and measurement of aortic size in patients with inherited aortopathies and in patients with suspected vasculitis), assessment of cardiac masses (including tissue characterization and evaluation of anatomic location), pericardial pathologies (including pericarditis), and congenital heart disease [19], [90]. These additional pulse sequences may be added as part of a comprehensive CMR protocol to adjudicate a secondary clinical question while addressing the primary conditions (e.g., arrhythmia, scarring, perfusion, cardiomyopathy).

## 8 Electrophysiology indications for CMR

Compared with CIED patients with suspected ischemic and non-ischemic cardiomyopathies, fewer CIED patients are indicated for VT or AF ablation.

### 8.1 Ventricular arrhythmias

• *CMR-based risk stratification*
  Late gadolinium enhancement (LGE) adds substantial value to current models predicting the risk of life-threatening cardiac arrhythmias and sudden cardiac death – particularly in patients with non-ischemic cardiomyopathies [91], [92], [93], [94], [95], [96] and patients with ventricular arrhythmias in the setting of preserved ejection fraction [97], [98], [99], [100].
• *CMR-aided ablation of ventricular arrhythmias*
  In patients with ventricular arrhythmias, LGE is frequently used for procedural planning and guidance of ablation procedure. While various periprocedural imaging modalities other than CMR can be used to assess cardiac function (e.g., echocardiography), obtain high resolution anatomy of the ventricles and extracardiac structures (e.g., CT) or rule out intracardiac thrombi (e.g., transesophageal or intracardiac echo, CT), LGE is the most proven clinically established non-invasive imaging method to determine tissue characteristics and arrhythmogenic substrate.
  LGE not only discriminates scar from healthy tissue, with the aid of 3D-reconstruction based on quantification of local relative signal intensities, it can also identify viable myocardium with heterogeneous electrophysiological properties within areas of dense scar. It is those “border zones“ defined by intermediate relative signal intensities, that typically harbor the arrhythmogenic substrate in terms of scar-pervading channels of slow conduction [101], [102], [103]. LGE-based assessment of arrhythmogenic substrate has been extensively validated. CMR-detected channels have been shown to predict future ventricular arrhythmia events [104], [105], and several studies demonstrated that CMR-guided ablation can reduce procedure times and improve clinical outcome [104], [105], [106]. It is noteworthy that potentially arrhythmogenic channels can be reliably detected by CMR also in CIED patients using specific wideband sequences avoiding hyperintensity artifacts, even in the proximity of the CIED [56], [107], [108].
  Ventricular tachycardia (VT) ablation can be performed without preprocedural CMR with LGE. However, insights from systematic endo- and epicardial mapping studies using high density mapping systems in recent years have fostered our awareness of the three-dimensionality of the arrhythmogenic substrate that can be augmented by 3D imaging modalities [109]. Even with combined endo- and epicardial approaches, electroanatomical mapping is confined to two dimensions and has limited specificity for detection of intramural substrates or substrate components. For instance, radiofrequency ablation lesions reach a depth of 0-3 mm or possibly 5 mm depending on the degree of catheter contact with the myocardium. Hence, if the area of LGE is located in the epicardium, an endocardial ablation approach is unlikely to reach the epicardial arrhythmogenic substrate if the myocardial wall is about 10 mm thick. Similarly, an intramural septal substrate where the area of LGE is confined to the midmyocardial septum, may be reachable neither from the left nor the right ventricular septum. If, however, the scar is predominantly endocardial, as in patients with prior myocardial infarction, an endocardial ablation procedure will be sufficient to target and eliminate the arrhythmogenic substrate. Bogun et al. [110] demonstrated successful elimination of arrhythmogenic substrate in a series of patients with non-ischemic cardiomyopathy by using different ablation approaches based on the location of the areas of LGE. The authors showed that the ablation procedure eliminated the ventricular arrhythmias with an endocardial approach when LGE was confined to the endocardium, and likewise, the procedure eliminated the ventricular arrhythmias with an epicardial approach when LGE showed an epicardial location. Either, endocardial or epicardial approach often failed in patients with an intramural substrate. The value of CMR in planning ablation procedures was also supported by others [111] and is the current clinical practice supported by expert consensus statements [112].
• *Identification of a deeper-seated substrate out of reach of ablation lesions*
  Intramural substrate is the most challenging scar distribution with respect to ablation outcome. In a small series of patients with nonischemic cardiomyopathy, an intramural substrate was associated with failed ablation procedures [110]. Furthermore, Ghannam et al. demonstrated that patients with nonischemic cardiomyopathy and deeper seated intramural scarring often have unsuccessful ablation procedures with conventional catheter technology [113]. The scar depth index was found to be larger in patients with failed ablations and VT recurrences. It is a measure of the amount of scar located at a depth > 5 mm (radiofrequency ablation lesions typically do not reach that deep) defined as the percent of scar at a depth > 5 mm projected to the closest endocardial or epicardial surface. A cut-off value of 17% scar was associated with ablation failure. Being aware that a particular patient has large regions of midmyocardial scarring sandwiched into thick myocardial tissue without LGE indicates that an ablation with conventional catheter technology is likely to fail to eliminate all ventricular arrhythmias and one should be prepared to use technology that has the potential to reach deep into the myocardial tissue.
  The specific substrate localization is a key determinant of success rates and procedural risk, with ablation of intramural substrates being particularly complex and epicardial access being associated with substantially elevated complication rates. Of note, LGE is capable of 3-dimensional localization of the arrhythmogenic substrate and in combination with CMR-based local wall thicknesses assessment, can also determine substrate accessibility with either an endocardial or epicardial approach [111]. Clinical benefits of procedural planning based on LGE to a priori determine ablation targets and the need and feasibility of an epicardial access have been demonstrated previously [110], [111], [114].
  The increasing acknowledgement of these benefits is reflected by the fact that LGE imaging has become part of the routine clinical workflow for ventricular arrhythmia ablation in many specialized centers.
• *Ventricular redo ablation lesion assessment*
  As LGE can also detect ablation-induced scarring, several studies have suggested CMR-based ablation lesion assessment for risk stratification and to guide treatment decisions in patients that have undergone ablation of ventricular arrhythmias [115], [116], [117], [118]. Mainly, ablation lesions correspond to areas of coagulative necrosis [119] and appear as dark core areas in patients with prior myocardial infarctions or nonischemic cardiomyopathy. Ablation lesions are not uniform and most likely depend on the degree of catheter contact at the time of the index ablation procedure. Ventricular arrhythmias often recur post ablation and repeat ablation procedures are required to eliminate recurring VT. The location of effective ablation lesions from prior procedures can be assessed by CMR and can give the operator an idea in conjunction with information from the available ventricular arrhythmias, whether the ventricular arrhythmia is a new arrhythmia or an arrhythmia that was previously ineffectively targeted. In the latter case, an alternative ablation approach may be required. Ghannam et al. further demonstrated that ablation lesions also can change the arrhythmogenic substrate and form borders for new or modified reentry circuits that can be identified by the dark core lesions [120]. Therefore, knowledge of the location of ablation lesions can expedite repeat mapping/ablation procedures by focusing on areas adjacent to ablation lesions that may be critical for a changed arrhythmogenic substrate.
• *Technical challenges for LGE in CIED patients undergoing VT ablation*
  Standard LGE pulse sequences are likely to yield low diagnostic yield due to severe image artifacts induced by the generator of CIEDs, resulting in “hyperintense” artifacts which may obstruct identification of myocardial scars [60], [121]. Wideband (segmented 2D [56], single-shot 2D [61], and 3D [108]) LGE would be preferred to suppress image artifacts. A recent study by Roca-Luque et al. demonstrated the value of 2D wideband LGE for guiding VT ablation in CIED patients [107]. Another technical challenge for scanning VT ablation candidates is the high burden of arrhythmias, which may result in ghosting artifacts in segmented 2D LGE and 3D LGE. In such patients, it may be preferred to perform wideband single-shot 2D LGE instead [61]. Finally, CIED patients with VT or ventricular fibrillation storm are at higher risk for CMR. In such patients, extreme caution should be exercised, and if scanning is warranted, the CMR protocol should be shortened to a bare minimum, possibly only performing LGE.

### 8.2 Atrial arrhythmias

• *LGE-based assessment of arrhythmogenic substrate*
  With long-term atrial arrhythmias recurrence rates up to 50% after catheter ablation, predictive tools to improve patient selection are needed. Particularly in patients with persistent forms of AF, recurrence rates are largely determined by the underlying arrhythmogenic substrate, often subsumed under the term atrial cardiomyopathy [122]. Fibrotic tissue remodeling defines distinct entities of atrial myopathies and is a key determinant of the arrhythmogenic substrate underlying atrial fibrillation. 3D left atrial (LA) LGE may detect atrial fibrosis, and the intensity of LGE correlates with the functional electrophysiological substrate in terms of reduced local conduction velocities [123].
  The seminal DECAAF trial in patients with no CIEDs, found 3D LGE to predict arrhythmia-free survival after catheter ablation and proposed risk stratification and treatment decisions based on the individual 3D LA LGE extent (UTAH-classification) [124]. However, to date such an approach has not been widely established due to deficits in spatial resolution of LGE for the left atrium and nonuniform definition and quantification of LGE, thereby resulting in insufficient reproducibility of the method [125]. Changes in fiber orientation takes place at the mid-myocardium and are not homogeneous across the atrium. Heterogeneity in fiber orientation is most prevalent at the roof, near the pulmonary veins, and at the inferior and anterior walls [126]. Anatomically, these areas comprise the intersection of major myocardial bundles such as the Bachmann bundle with oblique and circumferential bundles on the anterior left atrial wall. Interestingly, this mirrors the distribution of LGE in atria of patients with and without atrial fibrillation. Additionally, these regions with de novo LGE, which do not display low voltage, do display increased electrogram fractionation, which lends further support to varying conduction in distinct layers of myocardium with reduced interaction due to expanded interlayer spacing as identified by 3D LA LGE [127]. Furthermore, the DECAAF-II trial demonstrated that a CMR-guided approach for ablation of persistent atrial fibrillation was not superior to an approach without CMR guidance [128]. Therefore, additional studies to delineate the correlation of LGE in the myocardium with myocardial architecture and tissue composition are necessary before such regions are targeted with ablation [129]. To date, the value of 3D LA LGE has yet to be determined in patients with CIED.
• *Technical challenges for 3D left atrial LGE in CIED patients*
  The same challenges described for VT ablation applies here. Wideband 3D LA LGE would be preferred to suppress image artifacts induced by the generator of CIEDs.

### 8.3 Reduce fluoroscopy/procedure/anesthesia time/and improve outcomes

Knowledge of scar location can expedite ablation procedures in patients with structural heart disease by focusing the mapping procedure on areas with LGE [130], since LGE indicates location of arrhythmogenic substrate. This is the case for patients with prior myocardial infarctions and patients with nonischemic cardiomyopathy [110], [111]. Although large clinical trials are lacking, there is mounting evidence that preprocedural imaging with CMR helps to improve procedural outcomes [131].

## 9 Special considerations in pediatric patients and in patients with congenital heart disease

### 9.1 Anatomy and device placement

There are three major features that differentiate CIED management in children and have implications for CMR. First, congenital heart disease is a common substrate for arrhythmia disorders in children and young adults. Abnormal cardiovascular anatomy often requires non-MR-conditional systems and adds complexity to device care. Second, children are small and this alters the topology of heart, leads, and pulse generator; it also changes the long-term risks of permanent indwelling venous leads, skewing implant technique toward epicardial leads. Third, CIED indications typically persist for the remainder of each child’s life. Device planning must account for 50–80 additional years of device care. Each patient may experience multiple lead failures, lead extractions, and device revisions over a lifetime. Any exposure that could potentially speed along the next revision should be weighed carefully, including the rare elevations in thresholds that has been reported after MRI scanning.

**Fig. 4:**
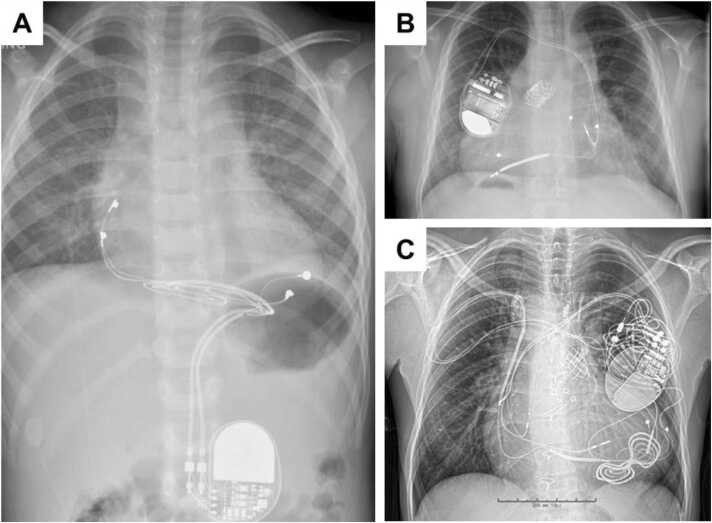
Chest radiographs from patients with congenital heart disease demonstrating a variety of non-MR-conditional CIED systems. (A) A dual chamber epicardial system placed in infancy. (B) A right-sided transvenous system placed in a patient with dextrocardia and a retained epicardial system. (C) A complex hybrid system after many years of CIED management, including bilateral transvenous systems, abandoned leads, an epicardial system tunneled to a subclavian pocket and an epicardial coil. Patients shown in (B) and (C) have an intravascular stent, unrelated to the pacing system.

Epicardial leads, sewn to the surface of the heart during a surgical procedure, are typically used for small children. In addition, patients with abnormal vasculature or intracardiac anatomy may require epicardial or hybrid systems. Lead failure is common during childhood [132], [133]. Thus, it is common for pediatric practices to follow children with epicardial systems, transvenous systems, and hybrid systems with complex device paths and abandoned leads (Fig. 4).

Epicardial leads and an abdominal pulse generator are the standard of care for infants and small children. The risk-benefit balance of endocardial vs. epicardial systems should be considered until children reach their full growth potential and some adults continue to have vascular access issues that preclude endocardial systems. We expect the incidence of new epicardial systems in children and adults with congenital heart disease to remain at similar levels for the foreseeable future. Epicardial leads are typically abandoned in place when the leads fracture or the device is moved electively to an endocardial position. The risks of a repeat sternotomy for lead extraction usually outweighs the benefits, except in the most pressing circumstances [133], [134] and lead fibrosis is often too dense to remove leads during repeat surgery for intracardiac palliation. Therefore, children with epicardial leads typically have lifelong retained leads. Importantly for imaging risk stratification, the status of abandoned epicardial leads (intact, capped or fractured) is often unknown to the imaging team at the time of MRI and cannot always be reliably assessed from a chest or abdominal radiograph. Leads that cannot be interrogated by an active pulse generator must be assumed to be fractured, a situation that has been associated in models with a higher theoretical risk of heating and adverse events [40], [43].

### 9.2 Changes in lead sensing and output threshold

To date, no permanent surgically implanted epicardial leads have been labeled as MR-conditional and models of epicardial systems have suggested that significant lead heating can occur [38], [43], [135]. However, epicardial leads have been scanned by MRI in many centers. To date, permanent clinical adverse events have not been reported as direct result of epicardial lead heating. In 2022, Vuorinen and colleagues published a case series on 17 patients with epicardial leads who received 26 MRIs [136]. One patient had a transient elevation of the ventricular pacing threshold in a chronic lead. A second patient had irreversible elevation of the atrial lead impedance, although the second event occurred six months after the scan and may have been unrelated. Other small series in pediatric patients have reported no adverse events, although the sizes of those series remain small: 5 to 40 patients [42], [137], [138], [139], [140]. A few larger series reporting primarily adult outcomes included a small number of pediatric patients [44], [135], [141].

### 9.3 Communication

In some implant configurations, there is a theoretical risk of lead tip heating of sufficient magnitude to cause cardiac damage, arrhythmia, or be detectable by the patient. Cardiac damage is covered elsewhere in this expert consensus statement; however, concerns for lead heating that causes detectable pain is important because a higher percentage of children require sedation or general anesthesia for MRI, compared to adults. A clinical complaint of sternal heating sufficient to cause patient discomfort was reported in 1 adult patient with a subcutaneous array in a study of 139 patients undergoing 200 MRIs [41]. In a pediatric study, 3 patients experienced mild discomfort at the CIED site during 54 CMR scans [42]. While none of these resulted in permanent harm, it is possible that without patient feedback, a subcutaneous coil or lead tip could heat sufficiently to affect cardiac or non-cardiac tissue and cause discomfort after re-awakening. While not all tissue damage causes symptoms, symptoms are an important feedback mechanism to warn of potential tissue damage. When possible, children should be sufficiently awake and aware to provide feedback to the scanning team. However, sedation and anesthesia are commonly required in pediatric patients. The absence of verbal feedback should be considered in the risk-benefit analysis. However, as a single risk factor, sedation or anesthesia usually does not add sufficient risk to withhold MRI imaging.

### 9.4 Image Quality

Image artifact from a relatively large CIED in a relatively small body can obscure clinically relevant information. For example, in a recent retrospective pediatric study, 9 of 54 cardiac MR studies (17%) had sufficient image artifact from the device itself that the study authors adjudicated the studies as “clinically useless” [42]. To date, none of the wideband CMR pulse sequences have been validated in pediatric patients. Prior to embarking on clinical imaging, MR physicians and treating physicians should consult to determine whether image artifact from the CIED is likely to obscure the critical diagnostic questions.

### 9.5 Summary of technical considerations for non-MR-conditional CIEDs in children and patients with congenital heart disease

**Fig. 5:**
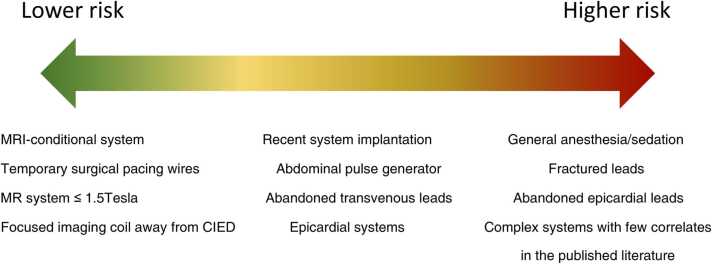
Spectrum of risk for MRI in pediatric and congenital heart disease patients with a CIED.

Children and patients with congenital heart disease are more likely to receive non-MR-conditional CIEDs than older adults with conventional anatomy. Table 7 summarizes expert consensus to date for MRI of pediatric patients with a CIED. Epicardial leads have higher theoretical risks of lead heating than endocardial leads and those risks are likely exacerbated by the presence of abandoned or fractured leads, both of which are common long-term sequelae of CIED management in this population. Even after transfer to a MR-conditional system, retained or abandoned leads may add risk to a patient in the MR environment. In addition, children are smaller and pulse generators are frequently implanted in the abdomen, near the ventricular mass, which increases the risk that image artifact obscures the diagnostic yield of CMR. However, these theoretical considerations are balanced by reassuring real-world data in this population. While the number of reported patients remains small, there has been no permanent morbidity directly attributable to exposure to the MR environment. Data extrapolated from adult studies suggests that many of non-MR-conditional CIEDs can be imaged safely. The decision to image non-MR-conditional devices requires placing the individual patient, CIED system, and MRI hardware/protocol along a continuum of risk (Fig. 5). The risks of the MRI scan should be balanced against the value of the diagnostic information that can be obtained and those risks and benefits should be communicated to the family, preferably with informed consent in writing as discussed in Section V.

**Table 7:** A summary of MR safety studies in pediatric patients with a CIED**.**

Pediatric-specific considerations	Citations	Summary of evidence
Patients of pediatric size require a pre-imaging assessment to ensure that image artifact is not likely to obscure diagnostic information.	[158], [159], [160], [161], [162], [163]	Strong evidence that CIEDs image artifact can prevent diagnostic quality imaging. Limited evidence suggests the problem is more prevalent in children.
A risk-benefit discussion, preferably with documented informed consent, should be obtained for imaging of non-MR-conditional systems or those with retained leads.	[42], [137], [138], [139], [140], [141]	With fewer than 100 pediatric patients in the literature, high-volume pediatric centers continue to obtain informed consent for non-MR-conditional systems
Sedation and general anesthesia increase the risks of undetected lead heating and should be avoided in children when possible	[41], [42]	Individual patients have reported discomfort during MRI scans, without objective evidence for harm.
Epicardial leads that cannot be assessed with an active pulse generator should be evaluated as if they were fractured.	[40], [43]	Lead fractures are not always obvious on chest radiography and fractured leads have been associated with a higher risk of tip heating.

## 10 CIED-like Heart Failure Devices

The rising burden of heart failure (HF) has led to innovations in device-based therapies, beyond traditional CIEDs, which aim to address the multidimensional aspects of HF pathophysiology including neuromodulation, respiratory dysregulation and volume overload [142], [143], [144]. Advent of novel HF devices poses specific MR safety considerations in this growing population. Select CE marked and FDA approved (Breakthrough Device Designation) devices are discussed (Table 8) along with MR safety.

**Table 8:** Overview of selected CIED-like heart failure devices.

Device	FDA approved device	Device specifics	MR Safety
Barorefelex activation therapy	Barostim Neo (CVRx, Inc., Minneapolish, MN)	Carotid sinus lead and subcutaneous pulse generator in ipsilateral chest.	MR Conditional for head, neck and lower extremity imaging
Phrenic nerve stimulation	remedē System (Zoll, Minnetonka, MN)	Transvenous phrenic nerve stimulation and sensing leads and subcutaneous pulse generator in chest.	MR Unsafe
Cardiac contractility modulation	Optimizer System, (Impulse Dynamics, Marlton, NJ)	Right ventricular pacemaker leads (2) and subcutaneous pulse generator in ipsilateral chest.	MR conditional at 1.5T for head and extremity imaging
Interatrial shunt devices	Corvia Atrial Shunt System (Corvia Medical, Inc., Tewksbury, MA) V-Wave (V-Wave Ltd., Caesarea, Israel)	Interatrial septal device creating a small left to right atrial shunt.	MR Conditional

Baroreflex activation therapy aims to treat autonomic dysregulation noted in HF by delivering electrical stimulation to carotid baroreceptors to restore autonomic balance [143]. The Barostim Neo (CVRx, Inc., Minneapolis, Minnesota) is FDA approved for symptomatic CRT-ineligible HF patients on optimal therapy [142] and has an MR-conditional safety label for head/neck and lower extremities exams [145]. The Barostim does not sense or respond to electrical activity and thus pauses therapy automatically during MRI scanning. Phrenic nerve stimulation aims to reduce sleep disordered breathing by treating central sleep apnea often seen in HF patients. The remedē System (Zoll, Minnetonka, Minnesota) is FDA approved but has been labeled as MR Unsafe and is contraindicated in patients known to require MRI [146]. Cardiac contractility modulation (Optimizer System, Impulse Dynamics, Marlton, New Jersey) uses electrical pulses to enhance contractility and targets intracellular calcium handing [143]. The Optimizer System, which is FDA approved for CRT-ineligible symptomatic HF patients on optimal therapy, has an MR-conditional label at 1.5T for head and extremity imaging, and requires programming prior to MRI scanning [147]. Interatrial shunt devices are designed to relieve left atrial pressure by shunting blood to the right heart. Several devices have been approved by the FDA (Supplemental Table 1) and carry the MR-conditional designation [148], [149].

The growing burden of HF has inspired innovative device-based therapies that continue to evolve. Safe and appropriate MRI scanning with these novel devices not only involves cognizance of the MR-safety label and artifacts, but also potential device-device interactions in patients with multiple implants (i.e., ICD and Optimizer).

## 11 Conclusion and future directions

This SCMR guideline statement outlines guidance on the following topics that are germane to delivering safe and effective CMR service to CIED patients. First, we summarized alternative imaging modalities for CIED patients. Second, we summarized the 2007 American Heart Association statement [15], the 2008 European Society of Cardiology statement [16], the 2017 HRS guideline [17], the 2021 recommendation by the International Society for Magnetic Resonance in Medicine safety committee [18], and the 2021 Canadian [19] and the 2022 British [20] societal consensus statements as the basis to build our document. Third, we described the requisite infrastructure, including legal/risk management, for starting a new CMR service for CIED patients with special attention to patient with non-MR-conditional CIEDs falling outside of the CMS coverage determination and 2017 HRS guidelines. Fourth, we summarized clinical indications not related to electrophysiology, including cardiomyopathies, infiltration, and ischemic heart disease. Fifth, we summarized clinical indications related to electrophysiology. Sixth, we described special considerations in pediatric patients and in patients with congenital heart disease, for which we have limited data. Seventh, we summarized key principles of MR physics describing MR safety, in particular the interaction between the RF field and intracardiac leads. This topic is of interest to vendors and researchers for developing improved strategies to further mitigate risk posed by CIED. Eighth, we summarized key strategies for pulse sequence optimization to improve image quality, which is important to increase benefit. Finally, we introduced emerging CIED-like heart failure devices based on limited data from the literature, given that patients with heart failure symptoms are likely to derive benefit from CMR [150].

Future studies include addressing safety for pediatric patients with epicardial leads, optimization and standardization of pulse sequences for CIED patients, optimization and standardization protocols in low-field (0.55T) and mid-field (3T) MRI scanners, and artificial intelligence or deep learning methods for predicting MR safety (risk), overreading image artifacts [151], and replacing image artifacts or signal voids with image inpainting [152].

## CRediT authorship contribution statement

**Nazarian Saman:** Conceptualization, Writing – original draft, Writing – review & editing. **Mont Lluis:** Conceptualization, Writing – original draft, Writing – review & editing. **Litt Harold:** Conceptualization, Writing – original draft, Writing – review & editing. **Zareba Karolina M.:** Conceptualization, Writing – original draft, Writing – review & editing. **Hu Peng:** Conceptualization, Writing – original draft, Writing – review & editing. **Manisty Charlotte:** Conceptualization, Writing – original draft, Writing – review & editing. **Patel Amit R.:** Conceptualization, Writing – original draft, Writing – review & editing. **Woodard Pamela K.:** Conceptualization, Writing – original draft, Writing – review & editing. **Lee Daniel C.:** Conceptualization, Writing – original draft, Writing – review & editing. **Rochitte Carlos E.:** Conceptualization, Writing – original draft, Writing – review & editing. **Hanneman Kate:** Conceptualization, Writing – original draft, Writing – review & editing. **Roguin Ariel:** Conceptualization, Writing – original draft, Writing – review & editing. **White James A.:** Conceptualization, Writing – original draft, Writing – review & editing. **Stojanovska Jadranka:** Conceptualization, Writing – original draft, Writing – review & editing. **Luetkens Julian A.:** Conceptualization, Writing – original draft, Writing – review & editing. **Collins Jeremy D.:** Conceptualization, Writing – original draft, Writing – review & editing. **Webster Gregory:** Conceptualization, Writing – original draft, Writing – review & editing. **Ng Ming-Yen:** Conceptualization, Writing – original draft, Writing – review & editing. **Kim Daniel:** Conceptualization, Writing – original draft, Writing – review & editing. **Mukai Kanae:** Conceptualization, Writing – original draft, Writing – review & editing. **Ennis Daniel B.:** Conceptualization, Writing – original draft, Writing – review & editing. **Davids Rachel:** Conceptualization, Writing – original draft, Writing – review & editing. **Bogun Frank:** Conceptualization, Writing – original draft, Writing – review & editing. **Weinsaft Jonathan W.:** Conceptualization, Writing – original draft, Writing – review & editing.

## Declaration of Competing Interest

The authors declare that they have no known competing financial interests or personal relationships that could have appeared to influence the work reported in this paper.

## Appendix

This section describes the interaction and safety considerations of the various required magnetic fields that are used during every MRI exam. Each of these fields interacts with a patient’s CIED in different ways, which leads to different safety considerations for each field. In general, there are two broad categories of concern – potential risks faced by the patient and the potential for damaging the CIED. A comprehensive review of these effects is provided by Panych and Madore [153]. A patient with an MR-conditional device can safely undergo an MRI exam when specific procedures are followed [154]. The safety and appropriateness of obtaining an MRI exam for a patient with a non-MR-conditional devices remains a topic of research [49].

### Static Magnetic (B_0_) Field

The MRI machine’s B_0_ field is required to generate the net polarization of spins needed to generate images. The strength of the B_0_ field is measured in units of Tesla (T) and the two mostly widely available MRI systems 1.5T and 3T fields. The B_0_ field is spatially uniform in magnitude (i.e., at isocenter there is very little spatial variation) and constant in time (i.e., it does not fluctuate).

One principal safety consideration for the B_0_ field include that it can exert pulling forces (strongest at the end of the scanner) or torque (at isocenter) on a CIED that contains ferromagnetic components. Modern MR-conditional CIEDs are generally free of ferromagnetic components, but this should be a consideration for a legacy non-MR-conditional CIED or pacing lead. There are also reports of “power on reset” (POR) for devices exposed to B_0_, but is very difficult to predict and may occur in the presence or absence of gradient and RF fields. This can deleteriously alter device programming [155], [156].

MRI-conditional FDA labeling for a specific device may list a maximum allowable spatial gradient of the static magnetic field (“spatial field gradient”) exposure limit in units of T per meter (T/m) [157]. The B_0_ field is spatially uniform at isocenter, but falls off substantially in strength at the ends of the scanner. As such, it is evident that the B_0_ field has an inherent spatial gradient in magnetic field strength (T/m). This B_0_ spatial gradient is always present and poses a potential safety risk – pulling or dislodging the device – especially when the patient’s CIED pass through the entrance of the scanner bore where the B_0_ spatial gradient is strongest.

This potential risk is present regardless of whether the MRI system is acquiring images or not since it only depends on the B_0_ field being “on”. Although the MR-conditional FDA labeling may specify a maximum allowable spatial field gradient exposure limit, it is oftentimes not known nor obvious what the B_0_ spatial field gradient is for each installed MRI scanner. This information may be obtained from the MRI system manufacturer or system installer. Note, it is important to discern the B_0_ spatial field gradient from the gradient system’s performance characteristics, for which the maximum gradient amplitude is defined in G/cm or T/m.

To avoid risks associated with exposure of a CIED to the B_0,_ it is necessary to compare the devices FDA labeling with the known characteristics of the MRI scanner’s B_0_ field and to only expose the CIED to the specific field strength identified in the FDA conditional labeling (1.5T and/or 3T). FDA conditional labeling at one field strength does not confer safety at a lower (or higher) field strength.

### Radiofrequency (RF) fields

The MRI machine generates transmit RF (B_1_ +) fields to, for example, excite spins into the transverse plane so that they generate a detectable signal for image formation. The applied RF-field for short durations (100 to 1000 microseconds), has a maximum amplitude of 10–30 µT, oscillates at the Larmor frequency (typically about 64 MHz at 1.5T or 128 MHz at 3T), and is shaped by an “envelope function” designed for a specific purpose (excitation, saturation, inversion, etc.).

A time varying magnetic field necessarily generates a complementary electric field (E-field) as a consequence of Maxwell’s equations. In general, the RF induced E-fields contribute to currents in conductive tissues that lead to resistive heating. Consequently, the allowable SAR or power deposited in the subject is regulated by the FDA because it contributes to patient heating. The specific RF fields needed for a particular MRI sequence can be characterized by either the SAR or the B_1_+rms (a measure of the time-averaged RF amplitude in units of µT). MR-conditional devices can carry labeling for either maximum SAR or B_1_+rms limits. SAR is estimated on the scanner and is patient specific, whereas B_1_+rms depends only on sequence parameters.

The principal safety consideration for the RF-field as it relates to CIEDs is that the induced E-field will generate a current in the pacing lead and power deposition at the lead-tip will result in lead-tip heating (LTH) that may damage tissue and or cause a loss of pacing capture [154]. The coupling of the RF E-field with the CIED is complex and depends on several variables. Some simple LTH variables have straightforward connections to LTH, but for complex LTH variables there is not an easily generalized contribution to LTH.

• *Simple LTH Variables*
  Both SAR and B1+rms are proxies for the potential for LTH. A higher SAR or B_1_+rms will contribute greater LTH. However, owing to the complexity of the interaction and range of devices there is no way to know how much LTH accords with a specific SAR or B_1_+rms. MR-conditional FDA labeling will provide a maximum allowable SAR or B_1_+rms exposure limit and each applied MRI sequence needs to be adjusted to meet this condition in order to keep LTH below acceptable limits. In addition, the RF-field is focused around isocenter, hence the risk of LTH is higher when the CIED is placed near isocenter, but falls off as the device moves away from isocenter. Patient orientation is another consideration and while it may not always be practical to obtain a CMR exam in a supine feet-first orientation, it has been identified as a potentially better orientation to limit LTH [48].
• *Complex LTH Variables*
  The interaction between the CIED and the RF induced E-field has a complex relationship to several variables. The amplitude and phase of the E-field is spatially varying and it interacts with the pacing lead that courses through this field along a patient-specific path and lead-length. The pacing lead effectively acts as an antenna picking up induced currents according with its interaction with the E-field. This gives rise to the lead-length and path dependence. In addition, the electronic characteristics of the lead will make it more or less receptive to the E-field. This E-field also oscillates at the Larmor frequency (different at 1.5T and 3T), which gives rise to the wave-length dependence of LTH. An antenna may be made more or less sensitive to receiving E-fields at a particular frequency, which CIED manufacturers use as a strategy to limit LTH in MRI-conditional devices. Unfortunately, the complexity of these interactions means that it is not easy to know if LTH will be worse at 1.5T or 3T, with a shorter or longer lead, or with a left or right-sided implant. Another potential concern is an abandoned [43] and epicardial leads [136].

Despite the complex relationship between the RF-field, the CIED, and the principal risk of lead-tip heating, it is possible to mitigate the risk principally by meeting the FDA conditional labeling of the CIED’s SAR or B_1_+rms limits. For legacy non-MR-conditional devices, MRI protocols can be adjusted to limit SAR and B_1_+rms exposure.

### Gradient fields

The magnetic field gradients are used for several purposes during an MRI exam including, for example, during spatial encoding. The magnetic field gradients are characterized by two magnetic field characteristics that define their performance. These include the maximum gradient amplitude measured in mT/m (typically 40–80 mT/m) and the maximum rate of change of the gradient amplitude, or slew rate, measured in T/m/s (typically 100–200 T/m/s). The magnetic field gradients are switched on and off rapidly during an MRI exam.

As with the RF-field a time varying magnetic field necessarily generates a complementary E-field. This E-field can, for example, induce currents in peripheral nerves leading to FDA limits on the gradient slew rate to mitigate peripheral nerve stimulation (PNS). Similarly, the gradients can induce currents in the pacing lead that can interfere with CIED rhythm sensing and contribute to oversensing or inappropriate therapy.

The interaction of the gradient induced E-field and the CIED lead path depends on the E-field characteristics, lead path, lead length, and lead type (bipolar vs. unipolar). As with RF-interactions, it is difficult to generalize the contribution to adverse interactions, but reduced gradient activity (lower slew rate) can mitigate the effects. To manage the risk of gradient fields interacting with the CIED the MRI exam should meet the FDA conditional labeling for the specific CIED and be programmed appropriately before the exam commences.
